# Machine learning-based prediction of motor status in glioma patients using diffusion MRI metrics along the corticospinal tract

**DOI:** 10.1093/braincomms/fcac141

**Published:** 2022-05-27

**Authors:** Boshra Shams, Ziqian Wang, Timo Roine, Dogu Baran Aydogan, Peter Vajkoczy, Christoph Lippert, Thomas Picht, Lucius S. Fekonja

**Affiliations:** Department of Neurosurgery, Charité - Universitätsmedizin Berlin, Klinik für Neurochirurgie mit Arbeitsbereich Pädiatrische Neurochirurgie, Campus Charité Mitte, Charitéplatz 1, 10117 Berlin, Germany; Cluster of Excellence: ‘Matters of Activity. Image Space Material’, Humboldt University Berlin, Berlin, Germany; Department of Neurosurgery, Charité - Universitätsmedizin Berlin, Klinik für Neurochirurgie mit Arbeitsbereich Pädiatrische Neurochirurgie, Campus Charité Mitte, Charitéplatz 1, 10117 Berlin, Germany; Department of Neuroscience and Biomedical Engineering, Aalto University School of Science, Espoo, Finland; Turku Brain and Mind Center, University of Turku, Turku, Finland; Department of Neuroscience and Biomedical Engineering, Aalto University School of Science, Espoo, Finland; Department of Psychiatry, Helsinki University and Helsinki University Hospital, Helsinki, Finland; A.I. Virtanen Institute for Molecular Sciences, University of Eastern Finland, Kuopio, Finland; Department of Neurosurgery, Charité - Universitätsmedizin Berlin, Klinik für Neurochirurgie mit Arbeitsbereich Pädiatrische Neurochirurgie, Campus Charité Mitte, Charitéplatz 1, 10117 Berlin, Germany; Digital Health - Machine Learning, Hasso Plattner Institute, University of Potsdam, Potsdam, Germany; Hasso Plattner Institute for Digital Health, Icahn School of Medicine at Mount Sinai, New York, NY, USA; Department of Neurosurgery, Charité - Universitätsmedizin Berlin, Klinik für Neurochirurgie mit Arbeitsbereich Pädiatrische Neurochirurgie, Campus Charité Mitte, Charitéplatz 1, 10117 Berlin, Germany; Cluster of Excellence: ‘Matters of Activity. Image Space Material’, Humboldt University Berlin, Berlin, Germany; Department of Neurosurgery, Charité - Universitätsmedizin Berlin, Klinik für Neurochirurgie mit Arbeitsbereich Pädiatrische Neurochirurgie, Campus Charité Mitte, Charitéplatz 1, 10117 Berlin, Germany; Cluster of Excellence: ‘Matters of Activity. Image Space Material’, Humboldt University Berlin, Berlin, Germany

**Keywords:** machine learning, support vector machine, tractography, diffusion MRI, corticospinal tract

## Abstract

Along tract statistics enables white matter characterization using various diffusion MRI metrics. These diffusion models reveal detailed insights into white matter microstructural changes with development, pathology and function. Here, we aim at assessing the clinical utility of diffusion MRI metrics along the corticospinal tract, investigating whether motor glioma patients can be classified with respect to their motor status. We retrospectively included 116 brain tumour patients suffering from either left or right supratentorial, unilateral World Health Organization Grades II, III and IV gliomas with a mean age of 53.51 ± 16.32 years. Around 37% of patients presented with preoperative motor function deficits according to the Medical Research Council scale. At group level comparison, the highest non-overlapping diffusion MRI differences were detected in the superior portion of the tracts’ profiles. Fractional anisotropy and fibre density decrease, apparent diffusion coefficient axial diffusivity and radial diffusivity increase. To predict motor deficits, we developed a method based on a support vector machine using histogram-based features of diffusion MRI tract profiles (e.g. mean, standard deviation, kurtosis and skewness), following a recursive feature elimination method. Our model achieved high performance (74% sensitivity, 75% specificity, 74% overall accuracy and 77% area under the curve). We found that apparent diffusion coefficient, fractional anisotropy and radial diffusivity contributed more than other features to the model. Incorporating the patient demographics and clinical features such as age, tumour World Health Organization grade, tumour location, gender and resting motor threshold did not affect the model’s performance, revealing that these features were not as effective as microstructural measures. These results shed light on the potential patterns of tumour-related microstructural white matter changes in the prediction of functional deficits.

## Teaser

Shams *et al*. report that glioma patients’ motor status is predicted accurately by diffusion MRI metrics along the corticospinal tract based on support vector machine method, reaching an overall accuracy of 77%. They show that these metrics are more effective than demographic and clinical variables.

## Graphical Abstract

**Graphical abstract:**
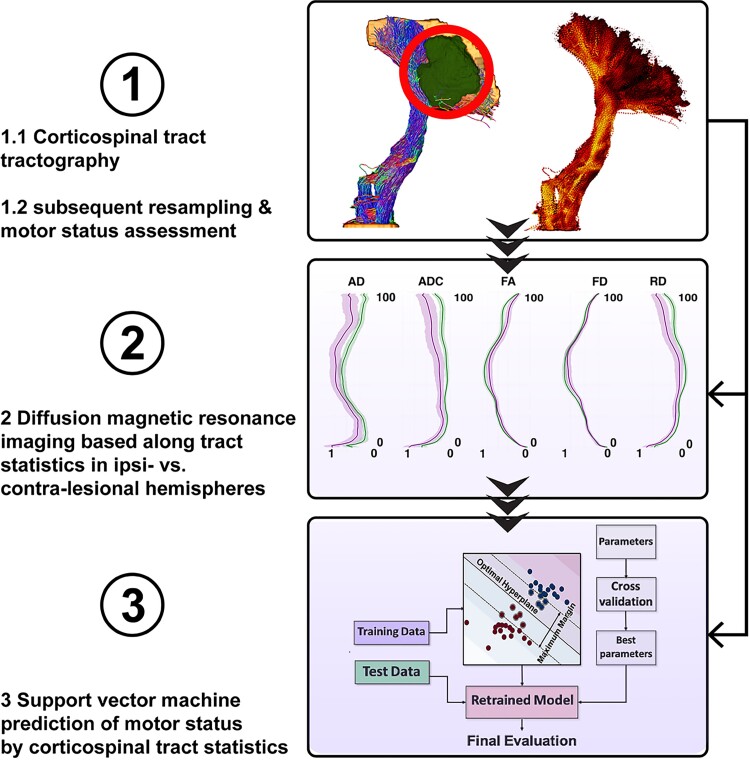

## Introduction

Gliomas are known as the most frequent and malignant human brain tumours, characterized by poor prognosis and high morbidity.^1^ Gliomas infiltrating the motor system potentially cause various degrees of damage to the white matter (WM) architecture and might lead to substantial motor function impairments.^1,2^ Diffusion MRI (dMRI)^3,4^ has shown potential by enabling non-invasive delineation of the WM pathways known as tractography^5–9^. Tractography has been frequently used for preoperative planning or analysing the effects of the tumour on WM and structural connectivity, for example, to investigate tumour infiltration and its impact on surrounding tissues.^10,11^

Along tract statistics enables WM characterization using various dMRI metrics.^2–4^. These measures have gained great interest since they reveal insights into WM development, function and disease.^15^ Along tract diffusion tensor imaging (DTI)-derived metrics such as apparent diffusion coefficient (ADC; a measure of the overall diffusivity), axial diffusivity (AD; the diffusion rate along the main axis of diffusion), fractional anisotropy (FA; the directional preference of diffusion) or radial diffusivity (RD; rate of diffusion in the transverse direction) and a more complex dMRI-based metric, namely fibre density (FD) have been previously used to study tumour-induced local microstructural changes.^16,17^ Here, we investigate whether along tract metrics can be used as predictive features to detect motor function impairments. Recently, we have shown that the segmental DTI-derived metrics, such as ADC and FA are associated with motor deterioration in patients with brain tumours.^18^ Multiple fibre populations are found in up to 90% of the WM voxels and 30–40% of these WM voxels contain more than three fibre populations.^19–22^ Moreover, non-WM contamination is found in more than a third of the WM voxels^23^ and multi-tissue constrained spherical deconvolution (CSD) methods^24–27^ have been used to account for it. As a result, CSD-based metrics in addition to DTI metrics (such as AD, ADC, FA or RD) are critical. By estimating fibre orientation distributions (FODs) in each voxel based on the expected signal from a single collinearly oriented fibre population, CSD can discriminate complex fibre populations.^28^ Probabilistic tractography algorithms, such as the iFOD2, have been proposed to overcome the limitations of tensor-based tractography methods by using the rich information in FODs.^29^ A complete picture of the underlying WM architecture is critical for risk assessment, neurosurgical planning and as well for prediction models.^30^ To that end, modern CSD-based FD and fixel-based analysis (FBA) approaches, in addition to traditional DTI methods, provide promising opportunities because they are related to the intra-axonal restricted compartment that is limited to a given fibre orientation within a voxel.^31,32^ More recently, we used FD for fibre orientation-specific study of dMRI properties along the tract in relation to infiltrating tumours,^16^ which was previously focused on group-based analyses.^31^ Yet, research lacks the individual and tract-specific characterization of WM microstructure investigating the association between tumour impact on structural connectivity and clinical assessment.

Machine learning (ML) methods have recently gained remarkable success in clinical applications such as diagnostic, prognostic and predictive analytics using various modalities of MRI scans.^33–36^

Here, we employed ML methods using along corticospinal tract (CST)-related dMRI metrics (e.g. AD, ADC, FA, FD and RD) to predict motor deficits in patients with motor-related glioma, focusing on individual diagnosis rather than GroupWise comparisons. We used ML methods based on support vector machines (SVMs) which is a powerful method, easy to interpret and well suitable method to handle large dimensional data sets.^37–39^ SVM has been used in various clinical applications such as tumour segmentation and classification, e.g. to distinguish low-grade gliomas from high-grade gliomas.^40–43^ In previous studies, glioma grading has been performed using resting-state functional MRI^44^ and radiomic.^45^ In addition, it has been used as a tool for non-invasive prediction of tumour consistency to classify the tumour as soft or firm for preoperative planning.^46^ Furthermore, it is known as an analysis tool for predicting the prognosis and survival time of tumour patients using multi-modal imaging.^35,47^ Considering our tumour patients’ cohort and multi-modal quantitative assessment of WM along CST based on dMRI metrics, we investigated our hypotheses using SVM-based analysis.

We designed our SVM models with an embedded feature selection method called recursive feature elimination (RFE)^48,49^ using histogram-based features of dMRI-based tract profiles. The histogram-based analysis is one of the most useful methods in many neuroimaging applications, e.g. classification and clustering tasks by which we can summarize and preserve more information from first-order statistics of the original data than simple averaging of the data values.^50,51^ A histogram model can be specified for a specific image feature type independently of any real image content.^50,51^

In addition, we developed an SVM model using principal component analysis (PCA)-derived^52,53^ components, regardless of histogram-based features. PCA transforms data from high-dimensional space (all segmental information of dMRI-based tract profiles) into a low-dimensional space.

Furthermore, patient demographics and clinical variables including the resting motor threshold (RMT), a transcranial magnetic stimulation (TMS)-derived neurophysiological marker, were incorporated and fed into our designed models. We then assessed the impact of all these features, e.g. demographics, clinical and microstructural features, on the performance of the model.

## Materials and methods

### Patient cohort

We included 116 left- and right-handed adult patients in this retrospective study (43 females, 73 males, average age = 53.51 ± 16.32, age range = 20–87). Only patients with an initial diagnosis of supratentorial, unilateral World Health Organization (WHO) Grades II, III and IV gliomas (16 WHO Grade II, 23 WHO Grade III and 77 WHO Grade IV) were included. All tumours were infiltrating or immediately adjacent to M1 and/or the CST either in the left or right hemisphere. Patients with recurrent tumours, previous radiochemotherapy or multilocular tumours were not included. The motor status was graded preoperatively according to the medical research council (MRC) scale for muscle power. Grade, 0 means no muscle power, and 5 means full muscle strength. All patients with MRC < 5 were assigned to the group with motor deficits (Class 1), and others (MRC = 5) were assigned to the group without motor deficits (Class 0).

### Image acquisition

Clinical MRI data were acquired preoperatively at Charité University Hospital, Berlin, Department of Neuroradiology over the past years, and data acquisition started before HARDI techniques were commonly used. The centre performed scans on 3 T Siemens Skyra scanner with dedicated 32-channel head/neck coil. The protocol included whole-brain high-resolution structural data, contrast-enhanced T1-weighted images, with TR/TE/TI 2300/2.32/900 ms flip angle = 9°, field of view = 256 × 256, 192 sagittal slices, 1 mm isotropic resolution, acquisition time: 5 min as well as a single shell diffusion-weighted volume with TR/TE 7500/95 ms, 2×2×2 mm^3^ voxels, 128 × 128 matrix, 60 axial slices, with 40 equally distributed orientations for diffusion-sensitizing gradients at *b*-value of 1000 s/mm^2^, for a total acquisition time of 12 min.

### Transcranial magnetic stimulation

Non-invasive functional motor mapping of both ipsilesional and contralesional hemispheres was performed in each patient using navigated TMS (nTMS) with NeXimia Navigated Brain Stimulation (Nexstim Oy, Helsinki, Finland). Each patient’s head was registered to the structural MRI and the composite muscle action potentials were captured by the integrated electromyography unit (sampling rate 3 kHz, resolution 0.3 mV: Neuroline 720, Ambu). The muscle activity (motor evoked potential, MEP amplitude ≥ 50 μV) was recorded by surface electrodes on the abductor pollicis brevis and first dorsal interosseous. Initially, the first dorsal interosseous hotspot, defined as the stimulation area that evoked the strongest MEP, was determined. Subsequently, the resting motor threshold, defined as the lowest stimulation intensity that repeatedly elicits MEPs, was defined using a threshold-hunting algorithm within the Nexstim eximia software. Mapping was performed at 105% resting motor threshold and 0.25 Hz. All MEP amplitudes > 50 μV (peak to peak) were considered as motor positive responses and exported in the definitive mapping.^54^ The subject-specific positive responses of the first dorsal interosseous were exported as binary 3 mm^3^ voxel masks per response in the T1 image space.

### Preprocessing and processing of MRI data

Preprocessing and processing of MRI data were performed as described earlier.^16^ Briefly, all T1 images were linearly (affine) registered to the dMRI data sets using advanced normalization tools (ANTs).^55,56^ Furthermore, we registered the human motor area template (HMAT) atlas to subject space with ANTs using the Symmetric normalization transformation model^55,57^ to obtain M1 seeding region of interests (ROIs).^55,57^ The preprocessing of dMRI data included the following and was performed within MRtrix3^58^ in sequential order: denoising,^59^ removal of Gibbs ringing artefacts,^60^ correction of subject motion,^61^ eddy currents^62^ and susceptibility-induced distortions^63^ in FSL,^64^ and subsequent bias field correction with ANTs N4.^65^ Each dMRI data set and processing step was visually inspected for outliers and artefacts. Scans with excessive motion were initially excluded based on a predefined threshold (if >10% outlier slices; however, this was not the case in the current cohort). We upsampled the dMRI data to a 1.3 mm isotropic voxel size before computing FODs to increase anatomical contrast and improve downstream tractography results and statistics.^66^ To obtain AD, ADC, FA and RD scalar maps, we first used diffusion tensor estimation using an iteratively reweighted linear least squares estimator, resulting in scalar maps of tensor-derived parameters.^3,67^ For voxel-wise modelling, we used a robust and fully automated and unsupervised method. This method allowed us to obtain three-tissue response functions for white and grey matter and cerebrospinal fluid from our data with the use of spherical deconvolution for subsequent usage in multi-tissue CSD-based tractography.^24,27,68^

### Tractography

Probabilistic multi-tissue tractography was performed based on the WM FODs with the iFOD2 algorithm^69^ as described earlier,^16^ with the slight modification of using the above-mentioned HMAT atlas-derived M1 seeding ROI.^70^ In brief, an inclusion ROI was defined in the medulla oblongata, tracking parameters were set to default with an FOD amplitude cutoff value of 0.1, a streamline minimum length of 5 × voxel size, and a maximum streamline length of 100 × voxel size. For each CST tractogram, we computed default *n* = 5000 streamlines per hemisphere. Each streamline per tractogram was resampled along its length to 100 equidistant points. Subsequently, we mapped AD, ADC, FA, FD and RD scalar metrics along the derived 100 equidistant points per streamline.

### Data preparation

We generated dMRI-based CST profiles, by which AD, ADC, FA, FD and RD were quantified at 100 segments along the CST using the values of the 100 points per streamline. To create a tract profile that is robust to outliers, we used two different methods and compared the results. In the first method, we calculated the median (Mdn) values across the 5000 streamlines per tractogram along its 100 segments. In the second method, we computed the segment-wise weighted mean (M) of the dMRI measures across streamlines. The streamline-wise contribution was weighted by the inverse Mahalanobis distance of the streamlines from the tract core (M). Streamlines that were more distant from M were considered less important.^15^ We used both ipsi- and contralesional CST profiles as input features (predictor variables); thus, the dimension of the imaging-based feature space was 1000 (5 metrics × 2 hemispheres × 100 segments = 1000 features).

### Statistical analysis

Statistical analysis and data visualization were carried out using Python 3.8.6. The main packages used were Scipy,^71^ Seaborn,^72^ Statsmodels^73^ and Matplotlib.^74^ To compare categorical variables, Fisher’s exact test (when the expected frequency was less than five per category) or Pearson’s χ^2^ test (for larger values) were employed. Two-tailed Student’s *t*-tests or Mann–Whitney U tests (Wilcoxon rank-sum test) were performed to compare continuous variables. Effect size (*r*) for Mann–Whitney U statistics was calculated as the *Z*-statistic divided by the square root of the number of samples. A significance level of *P* < 0.05 was considered as cutoff. With respect to multiple comparison analyses, statistically, significant *P*-values were false discovery rate (FDR) corrected using the Benjamini–Hochberg procedure.^75^ For all univariate statistical analyses, Mdn-based tract profiles were used. Canonical correlation analysis (CCA) was performed as a multivariate correlation analysis to identify and measure the association among all dMRI-based extracted features (both in ipsi- and contralesional hemispheres) and age.^76^ This analysis extracts meaningful information from a pair of data sets, dMRI-based features and age, by seeking pairs of linear combinations from two sets of variables with the maximum pairwise correlation. CCA was performed both on the patient’s cohort and on each patient’s group (Class 0; Class 1) separately. A more detailed analysis investigating the associations between each metric and age was also performed.

### SVM classification

**Figure 1:**
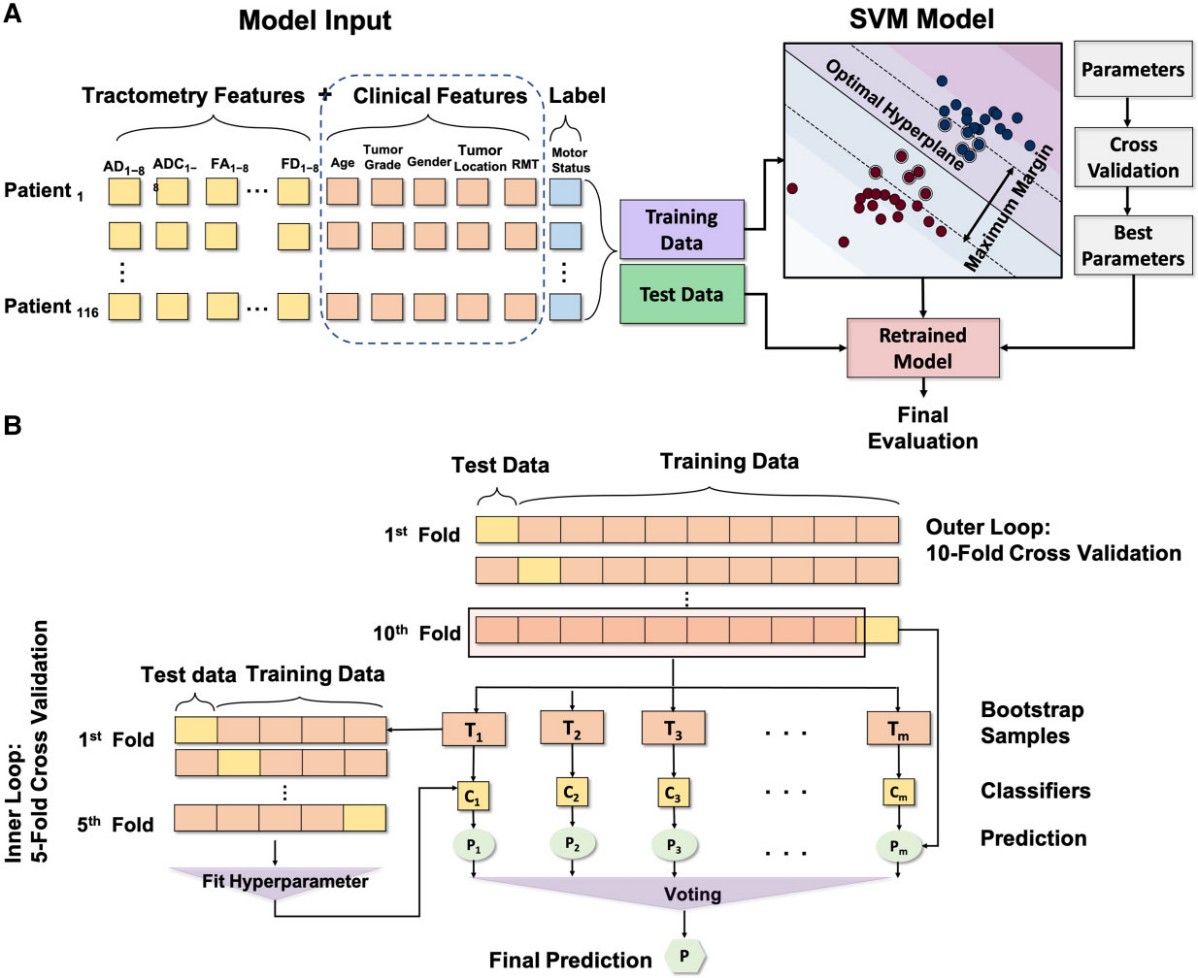
**Visual summary of the machine learning pipeline with nested cross validation and bootstrapping.** (**A**) dMRI-based extracted features are incorporated with demographic and clinical features and fed into the SVM model. The data set is split into a training and a test data set accordingly. (**B**) First, test and training sets are selected per fold of outer loop CV. Next, 1000 new synthetic data sets (*T_i_*) are generated from training set by randomly sampling from it. Hyperparameters of the classifiers (*C_i_*) are optimized within the inner loop. Finally, the estimator (*P_i_*) for each of the synthetic sets is found and the prediction (*P*) is voted across them.

SVM has gained a widespread application in the neuroimaging context as either classification or regression method.^77,78^ An SVM classifier aims to find hyperplanes with maximal margins between classes. As a supervised ML method, SVM can be extended to complex instances that are not linearly separable using so-called kernel tricks.^79,80^ Kernel techniques map input features from one space to a higher dimensional feature space in which different classes can be distinguished by a separating hyperplane. All ML analysis methods should be balanced between their predictive accuracy and descriptive power.^81^ Accordingly, in the present study, we developed different models based SVM method using dMRI-based features, demographic and clinical variables to predict the motor status (Class 0; Class1) preoperatively (cf. Fig. 1).

Some segments along tract profiles were missed (not-a-number value) when the tract profiles were generated. These missing values were imputed using two different interpolation methods^82^: (i) Mdn and (ii) k-nearest neighbour (KNN).^83^ Before fitting a model to our data, imputation of missing values and feature standardization were performed. To enable our classifier to learn from low and high variance metrics, we removed each feature’s M and scaled it to a unit variance (*z*-score). Training and test sets within each cross validation (CV) were standardized separately by M and standard deviation (STD) derived from the training set to prevent information leakage between testing and training data sets.

Four different SVM models were trained and tested using Mdn-based and Mahalanobis-based weighted M tract profiles with the above-mentioned interpolation methods for imputation of missing values (**SVM_1**: Mdn-based imputation method and Mdn-based tract profile; **SVM_2**: KNN-based imputation method and Mdn-based tract profile; **SVM_3**: Mdn-based imputation methods and Mahalanobis-based weighted M tract profile; **SVM_4**: KNN-based imputation method and Mahalanobis-based weighted M tract profile).

**Figure 2:**
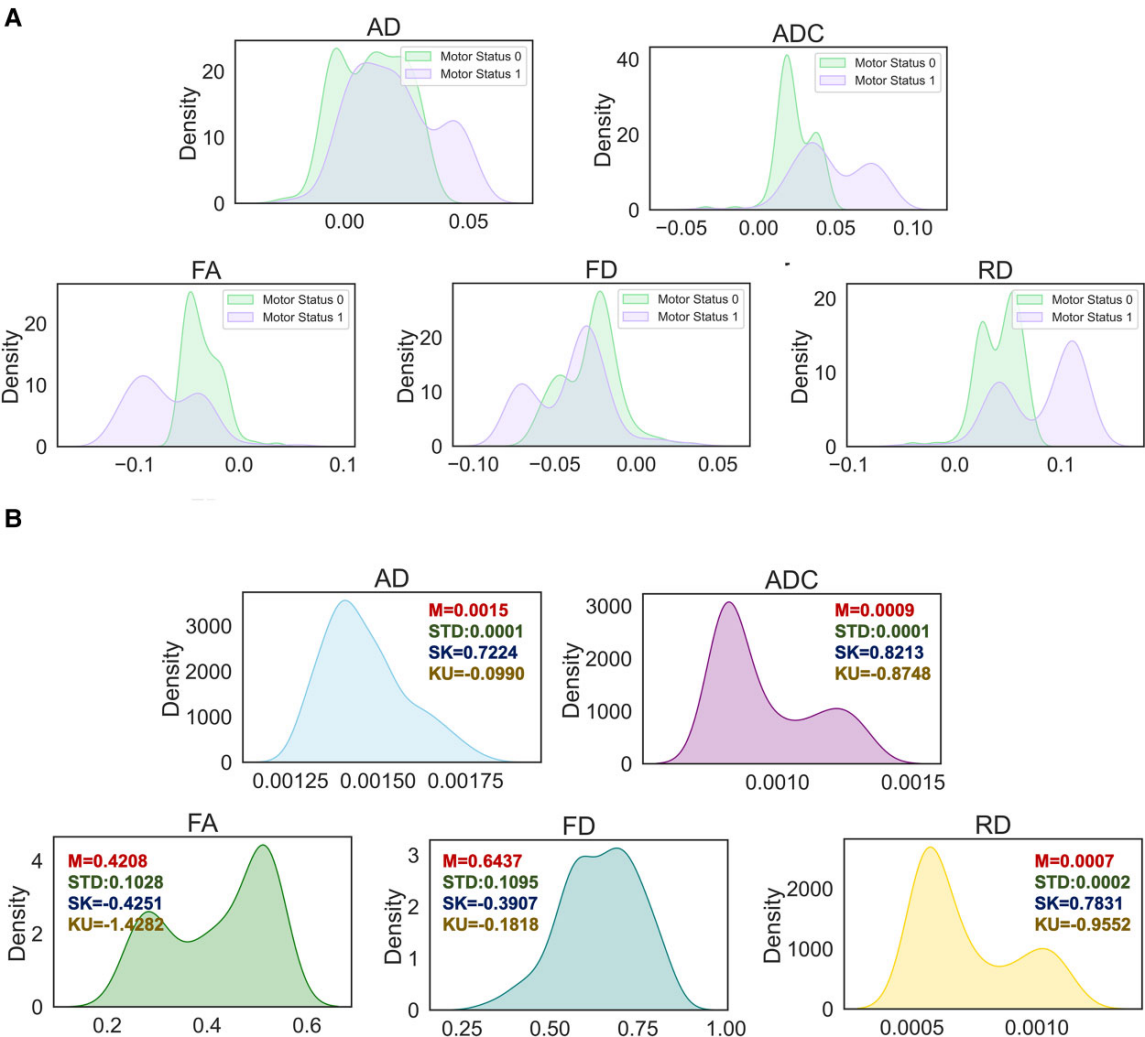
**Kernel density estimation (KDE) plot.** (**A**) Approximating the underlying probability density function based on KDE for dMRI-based ipsilesional CST profiles using mean values for patients with and without motor deficits; values are normalized for estimating kernel density. (**B**) KDE for a specific patient; a male patient (age: 81 years old) with preoperative motor deficits (Class 1) and glioma WHO Grade IV in the left hemisphere. Each plot is annotated with the corresponding histogram-based features (four features: M, STD, SK and KU).

As a preprocessing step, to reduce the high dimensional imaging-based feature space, a set of statistical features was calculated as a high-level representation to measure different properties of dMRI-based tract profiles’ distributions. Descriptive statistics such as M as a central tendency, STD as a measure of variability and kurtosis (KU), and skewness (SK) as measures of shape were extracted as histogram-based features (Fig. 2). The tract profile statistics were calculated for ipsi- and contralesional tractograms (4 measures × 5 metrics × 2 hemispheres = 40 features) and were fed into the models. We further incorporated patient demographics and clinical data such as age, gender, tumour grade, tumour location and RMT ratio, fed them into the aforementioned models, and compared the results.

The most relevant features were selected using the RFE method based on SVM (SVM-RFE).^48,49^ This method recursively removes features that contribute least to the prediction based on the linear SVM classifier weight coefficients before the actual learning phase. Subsequently, selected features were used to train and validate the SVM model with a linear kernel.

To investigate how well each dMRI metric (e.g. AD, ADC, FA, FD, RD) performed in classifying the patients with respect to their motor status, different SVM models were trained and tested, e.g. SVM_AD, SVM_ADC, SVM_FA, SVM_FD and SVM_RD, with KNN-based imputation method for missing values and Mdn-based tract profile (cf. Table 1). To assess the predictive power of patient demographics and clinical variables regardless of imaging-based features (when ignoring the neuroimaging analysis pipeline), an SVM model (SVM_clinical) was developed using only patients’ age, tumour WHO grade, tumour location, gender and RMT.

**Table 1:** The overview of all SVM models

Model	Input data	Imputation method for missing values	Method for generating the tract profile	PCA components	Histogram-based features
SVM_1	dMRI metrics	Mdn	Mdn	No	Yes
SVM_2	dMRI metrics	KNN	Mdn	No	Yes
SVM_3	dMRI metrics	Mdn	Weighted-mean	No	Yes
SVM_4	dMRI metrics	KNN	Weighted-mean	No	Yes
SVM_5	dMRI metrics	KNN	Mdn	Yes	No
SVM_AD	dMRI (AD metric)	KNN	Mdn	No	Yes
SVM_ADC	dMRI (ADC metric)	KNN	Mdn	No	Yes
SVM_FA	dMRI (FA metric)	KNN	Mdn	No	Yes
SVM_FD	dMRI (FD metric)	KNN	Mdn	No	Yes
SVM_RD	dMRI (RD metric)	KNN	Mdn	No	Yes
SVM_clinical	Clinical + demographic	No	No	No	No

Additionally, a model was developed using all values of ipsi- and contralesional dMRI-based tract profiles without performing above-mentioned feature extraction method. To reduce the high-dimensional imaging-based feature space (1000), PCA^52,53^ was performed on MdN-based tract profiles, and the first four components were fed into an SVM model with the linear kernel (SVM_5) using KNN-based imputation method (cf. Table 1).

We evaluated our models (SVM_1-5; SVM_AD-RD; SVM_clinical) using nested CV with a 10-fold outer loop and a 5-fold inner loop. Our model key hyperparameter C for penalty^84^ was optimized in the inner CV loop and the best performing model was applied to the outer CV loop test set to evaluate the model selected by the inner loop. C was tested from 0 to 10 with a 0.1 step size.

Our data set was imbalanced because the proportion of patients with motor deficits to patients without motor deficits was nearly 1 to 2 (cf. Table 2). We used a stratified 10-fold CV to ensure that class distributions in each data split matched the distribution in the complete training data set. We additionally assigned the class weights, *w*_*j*_ (Class 1: MRC < 0; Class 0: MRC = 5) inversely proportional to their respective frequencies as $w_{j}=\frac{n_{\mathrm{samples}}}{n_{\mathrm{classes}}*\;n_{\mathrm{samplesj}}}$, where *n*_samples_ is the number of samples, *n*_classes_ is the number of classes and *n*_samples*j*_ is the number of samples per class.

**Table 2:** Demographic and neuropathological overview of the patient cohort

	Patients without motor deficits Class 0	Patients with motor deficits Class 1	*P*-value
*Demographics*			
Cohort size	71 (62%)	45 (37%)	_
Age	50.25 ± 15.85	58.64 ± 15.45	0.005
Female	25 (35%)	18 (41%)	0.63
Male	47 (65%)	26 (60%)	0.63
*Ipsilesional hemisphere*			
Left	30 (42%)	17 (39%)	0.77
Right	41 (57%)	28 (64%)	0.77
*Tumour location*			
Frontal	35 (49%)	25 (55%)	0.55
Parietal	19 (27%)	12 (27%)	0.91
Insular	10 (14%)	5 (11%)	0.77
Temporal	7 (10%)	3 (7%)	0.73
*Glioma WHO grade*			
II	13 (18%)	3 (7%)	0.8^a^, 0.05^b^
III	18 (25%)	5 (11%)	0.8^a^, 0.03^c^
IV	40 (55%)	37 (84%)	0.05^b^, 0.03^c^
RMT* (V/m)			
Ipsilesional hemisphere	33.72 ± 7.2	34.64 ± 9.15	0.57
Contralesional hemisphere	34.3 ± 6.05	35.62 ± 8.64	0.37

*Resting Motor Threshold (RMT), TMS-derived neurophysiological marker, indicating cortical excitability

^a^ WHO Grade II versus III.

^b^ WHO Grade II versus IV.

^c^ WHO Grade III versus IV.

Bootstrap aggregating (bagging) has been introduced as a method to reduce the variance of a given estimator.^85^ Bagging involves applying an estimator to multiple bootstrap samples and voting the results across them. These estimators can use CV themselves to select fine-tuning parameters trading off bias and variance of the bootstrap sample-specific candidate estimators. We used this approach in our models (SVM_1-4; SVM_AD-RD) with 1000 resampled training sets per fold of outer loop CV and lastly voted among all 1000 generated models.

We evaluated the performance of our models using the overall accuracy, the ratio of correctly predicted samples over the entire cohort, sensitivity, specificity and the area under the receiver operating curve (AUC).

We used various SVM models to predict tumour-related motor deficits. To this end, four SVM models (SVM_1-4) were trained using histogram-based features of dMRI-based CST profiles, and one SVM model (SVM_5) was trained using four PCA components of segmental information of dMRI-based CST profiles. Finally, an additional SVM was trained (SVM_Clinical) using clinical and demographic features.

### Data availability

Raw data that support the findings of this study are not publicly available due to information that could compromise the privacy of the research patients. However, the code we have used is openly available on https://github.com/CUB-IGL/Machine-learning-based-prediction-of-motor-status-in-glioma-patients-using-dMRI-metrics-along-CST.git and is referred to at the corresponding passage in the article.

## Results

Forty-five (37.9%) of the recruited 116 patients presented with preoperative motor deficits (MRC < 5; cf. Table 2). There were no significant differences in gender (χ^2^[1, *N* = 116] = 0.51, *p* = 6.3*e* − 1) or hemispheric pathology position (χ^2^[1, *N* = 116] = 0.08, *p* = 7.7*e* − 1) in relation to motor deficits. Patients with motor deficits (Class 1) were older (58.64 ± 15.45) than patients without motor deficits (Class 0) (50.25 ± 15.85), with a highly significant difference between them (*t*[114] = 2.83, *p* = 5*e* − 3), although a medium effect was found (g=5.3e−195%CI=[0.155−0.914]). There were no significant differences in tumour locations and RMT ratio in both ipsi- and contralesional hemispheres in relation to motor deficits (cf. Table 2). We also found significant difference between the glioma WHO Grades III and IV between patients with and without motor deficits (χ^2^[1, *N* = 116] = 0.86, *p* = 3*e* − 2).

Moreover, considering CCA, we found a strong positive correlation between all dMRI-based extracted features and age, which was statistically significant (*r*_*s*_(114) = 0.7, *p* = 3.3*e* − 18). The correlation coefficients for the CCA are provided in Supplementary Table 1. This correlation was stronger in the group with motor deficits (Class 1; *r*_*s*_(45) = 0.93, *p* = 6.12*e* − 21). Considering each metrics separately, the correlation was weaker (**AD:***r*_*s*_(45) = −0.59, *p* = 1.64*e* − 5; **ADC:***r*_*s*_(45) = 0.64, *p* = 2.32*e* − 6; **FA:***r*_*s*_(45) = −0.42, *p* = 3.2*e* − 4; **FD:***r*_*s*_(45) = −0.24, *p* = 0.1; **RD:***r*_*s*_(45) = 0.51, *p* = 3.54*e* − 4). In the group without motor deficits (Class 0), the dMRI-based extracted measures were negatively correlated to age (*r*_*s*_(71) = −0.89, *p* = 1.0*e* − 25). Considering each metrics separately, the correlation was weaker (**AD:***r*_*s*_(71) = 0.33, *p* = 4.0*e* − 4; **ADC:***r*_*s*_(71) = 0.53, *p* = 2.07*e* − 6; **FA:***r*_*s*_(71) = −0.49, *p* = 1.64*e* − 5; **FD:***r*_*s*_(71) = −0.55, *p* = 4.87*e* − 7; **RD:***r*_*s*_(71) = 0.58, *p* = 1.2*e* − 7).

### Group-wise statistical analysis

**Figure 3:**
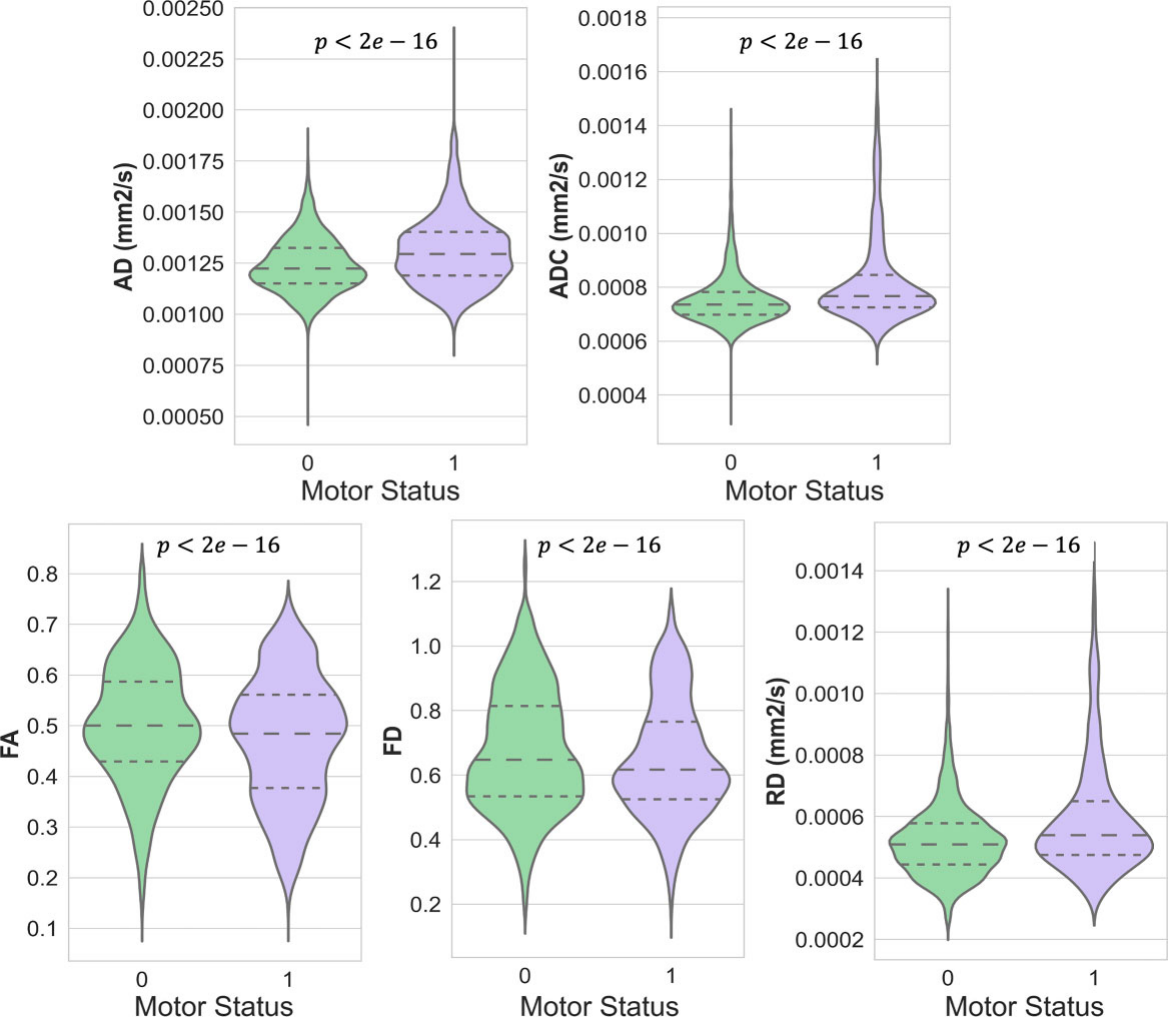
**Violin plots.** Violin plots are illustrating the frequency distribution of all segmental values related to AD, ADC, FA, FD and RD metrics over the ipsilesional CST. *P*-values were calculated using a two-tailed Student’s *t*-test (*n* = 100*71, Class 0, without motor deficit, MRC = 5; *n* = 100*45 Class 1, with the motor deficit, MRC < 5); horizontal lines indicate median and quartiles.

A comprehensive group-wise analysis was performed over the entire CST and segment-wise along the 100 segments to compare the differences in dMRI metrics between the two patient groups with (Class 1) and without (Class 0) motor deficits. The violin plots in Fig. 3 show significant differences in dMRI-based measures in the ipsilesional CST between the two patient groups.

**Figure 4:**
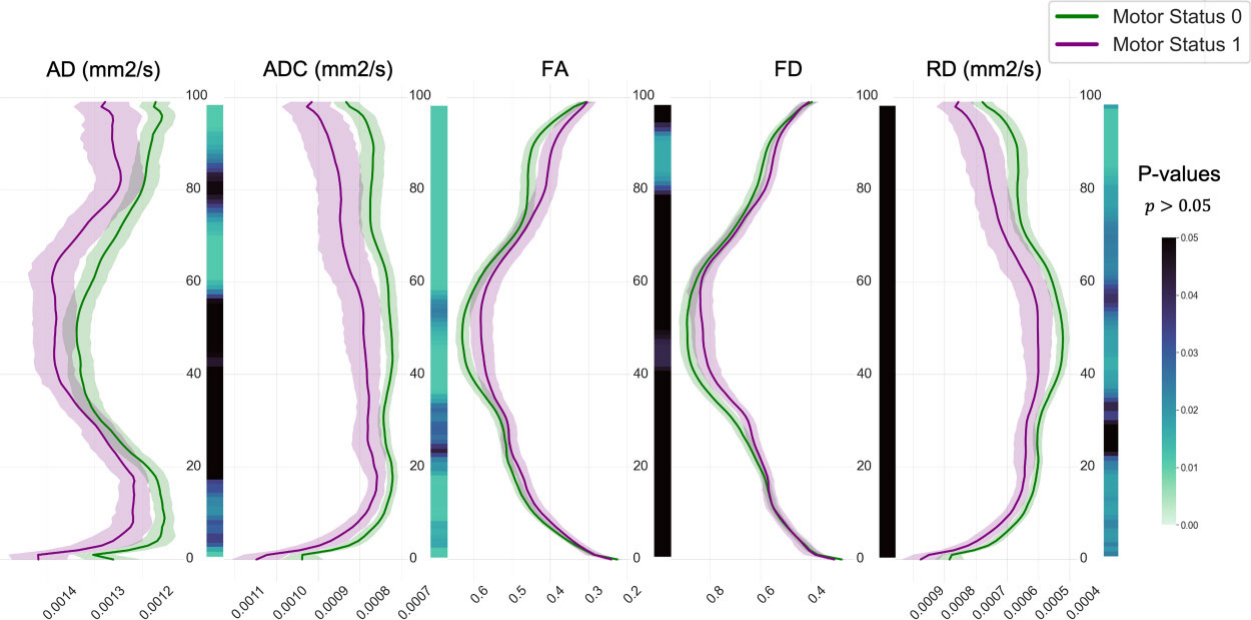
**Line plots.** Line plots are illustrating AD, ADC, FA, FD and RD metrics along the ipsilesional tractogram (segment 0 = medulla oblongata; segment 100 = M1), for both motor patients’ groups (*n* = 71, class 0, MRC = 5; *n* = 45, class 1, MRC < 5). The lines indicate median values with their 95% confidence interval. The heatmaps demonstrate related FDR corrected *P*-values, as tested with a two-paired Student’s *t*-test.

Figure 4 shows a segment-wise comparison of dMRI metrics between the two groups of patients (Class 0; Class 1) in ipsilesional CST. We found significant segment-wise differences, surviving FDR correction, between the two groups in the ipsilesional CST profiles in relation to ADC, AD, FA and RD metrics. However, no significant segment-wise differences were found with respect to FD (cf. Supplementary Table 2). These differences were larger in ADC and RD, especially in the tracts’ middle and peritumoural areas. The ipsilesional tract profiles led to larger significant differences compared with the differences between ipsi- and contralesional CSTs in group analyses (cf. Supplementary Table 3). In the latter case, only 6 nodes (93–98th) in the ADC, 9 nodes (88–96) in FA and 11 nodes (88–98) in RD metrics of the ipsilesional tracts showed significant differences between the two groups (class 0; class 1) mainly at the superior portion (area between the cortex and the internal capsule) of the CST. Moreover, the significant segments in contralesional tract profiles were only seen in a few segments in AD and ADC tract profiles (cf. Supplementary Table 4).

**Figure 5:**
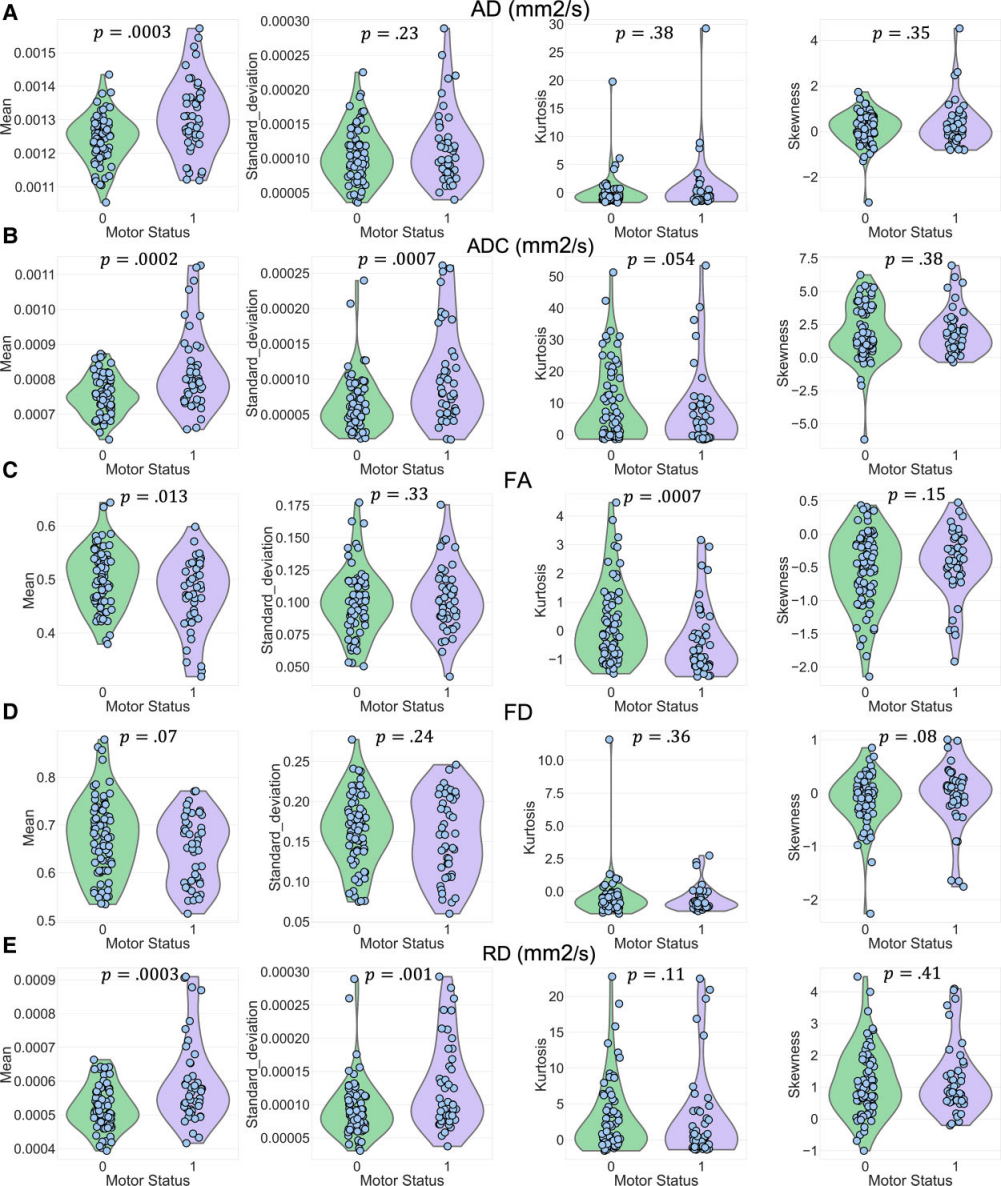
**Violin plots.** Violin plots are illustrating different histogram-based measures of ipsilesional CST profiles based on (A) AD (mm^2^/s), (B) ADC (mm^2^/s), (C) FA, (D) FD and (E) RD (mm^2^/s) metrics in in two patient groups (*n* = 71, Class 0, MRC = 5; *n* = 45, class 1, MRC < 5). The analyses were done using Mann–Whitney U-tests.

Additionally, we used M, STD, KU and SK as histogram-based measures of tract profiles and performed group-wise analyses on each measure for ipsi- and contralesional tract profiles (cf. Supplementary Table 5). As shown in Fig. 5A–E, the M measure over the ipsilesional CST profiles of AD, ADC, FA and RD was significantly different between the two patient groups with and without motor deficits (**AD:***U* = 1000, *p* = 3.6*e* − 4, *r* = 0.37; **ADC:***U* = 974, *p* = 2.07*e* − 4, *r* = 0.4; **FA:***U* = 1214, *p* = 1.5*e* − 2, *r* = 0.24; **RD:***U* = 1003, *p* = 3.8*e* − 4, *r* = 0.42) as well as the KU measure of ipsilesional CST profile of FA value (*U* = 1034, *p* = 7.1*e* − 4, *r* = 0.35). Further in Fig. 5B and E, STD of ADC and RD profiles showed a highly significant increase in the patient group with motor deficit (**ADC:***U* = 1032, *p* = 6.8*e* − 4, *r* = 0.35; **RD:***U* = 1065, *p* = 1.2*e* − 3, *r* = 0.33).

Ipsilesional tract profile measures led to larger differences compared with the contralesional tract profile measures. Nine ipsilesional tract profile measures (extracted features) were significantly different between the two patient groups (see Fig. 5), while only three measures of contralesional tract profiles were significantly different (cf. Supplementary Table 5).

### SVM classification

SVM_clinical, using all patient demographics and clinical variables as input features, received a low performance score (58% accuracy, 82% sensitivity, 43% specificity and 62% AUC). Among all SVM_AD-RD models, when using microstructural measures in relation to each metric separately, SVM_FA reached the highest accuracy (67%), sensitivity (60%) and AUC (68%), and SVM_ADC and SVM_RD reached the highest specificity (80%). The AUC in SVM_ADC and SVM_AD reached nearly the same score (67%, Fig. 4).

Considering SVM_1-4 models, using all measures of dMRI-based tract profiles, the best classifier performance was achieved with SVM_2, which reached 74% accuracy, 74% sensitivity, 75% specificity and 77% AUC (Table 3). With the KNN interpolation method, the number of nearest neighbours when K = 10 yielded the best model performance.

**Table 3:** SVM_1-4 model performances using Mdn versus KNN interpolation methods and Mdn versus Mahalanobis-based weighted mean tract profile

	Accuracy (%)	Sensitivity (%)	Specificity (%)	AUC (%)
*Mdn-based profile*				
Mdn interpolation (SVM_1)	73	73	73	76
KNN interpolation (*k* = 10) (SVM_2)	**74**	**74**	**75**	**77**
*Weighted M profile*				
Mdn interpolation (SVM_3)	68	66	70	70
KNN interpolation (*k* = 10) (SVM_4)	69	66	70	70

The five most effective features that were selected by SVM-RFE are FA_KU, FA_SK, RD_KU, ADC_STD and ADC_M; effect sizes and FDR-corrected *P*-values were also calculated (**FA_KU:***U* = 1034, *p* = 1.1*e* − 3, *r* = 0.35; **FA_SK:***U* = 1413, *p* = 1.5*e* − 1, *r* = 0.11; **RD_KU:***U* = 1377, *p* = 1.3*e* − 1, *r* = 0.13; **ADC_STD:***U* = 1032, *p* = 1.1*e* − 3, *r* = 0.35; **ADC_M:***U* = 974, *p* = 1.0*e* − 3, *r* = 0.4). Table 4 shows these selected features with their respective learned weights using SVM_2.

**Table 4:** SVM2 selected features with their respective learned weights using SVM-RFE

Features	Weights
FA_KU	1.29
FA_SK	1.17
RD_KU	1.07
ADC_STD	1.01
ADC_M	0.61

**Figure 6:**
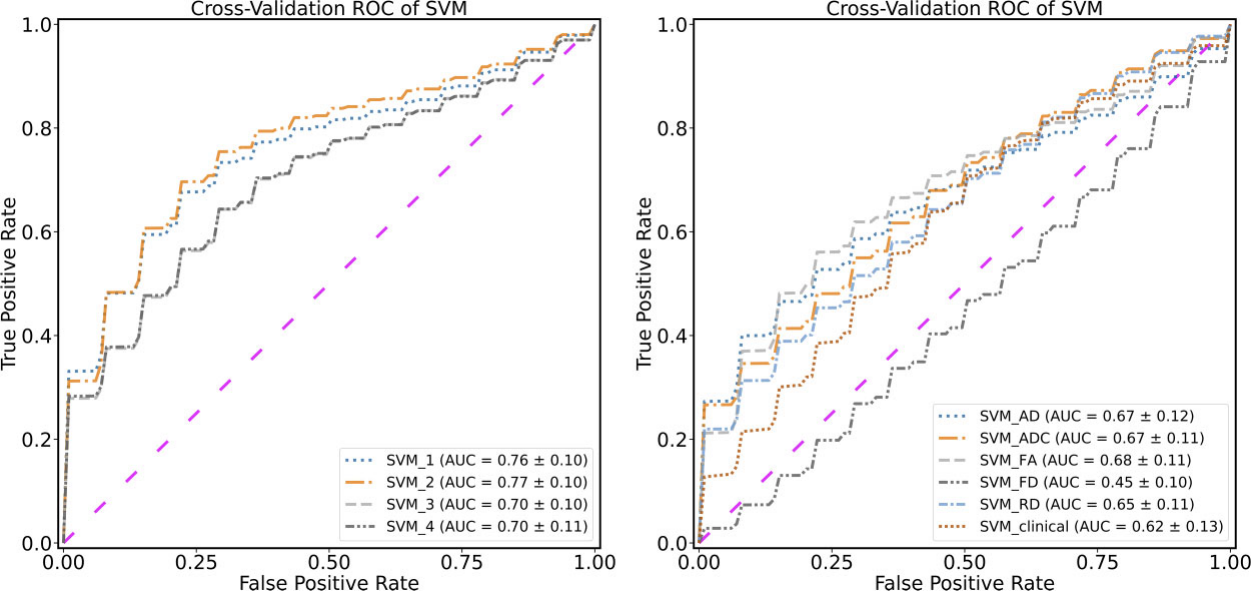
**Receiver operating characteristic (ROC) curves.** ROC of the discriminative performance of the SVM_1-4 and SVM_ADC-RD models show the true positive rate against the false-positive rate for different thresholds in comparison to classifiers with a random performance level (diagonal, dashed line).

In the last step, patient demographics and clinical features were integrated into our models (SVM_1-4). The feature selection method did not select them in any of the trained models (SVM_1-4) and the models’ performances remained unchanged. The receiver operating characteristic curves for SVM_1-4, SVM_AD-RD and SVM_clinical are presented in Fig. 6.

Moreover, we developed a model using all segment-wise information along ipsi- and contralesional tract profiles for all dMRI metrics (ADC, RD, AD, FA and FD) with PCA (SVM_5). This model reached 63% accuracy, 64% sensitivity, 63% specificity and 70% AUC. Summarizing the SVM results, we showed that the best model performance was achieved when we generated a tract profile based on median values and imputed the missing values with the KNN method (SVM_2). Moreover, our model that included only clinical and demographic features (SVM_clinical) was less accurate than the models with microstructural measurements. All models were trained separately using each modality (SVM_AD-RD) and were also less accurate than other models that included imaging features from all modalities (SVM_1-4). Additionally, our SVM model, which used PCA components, performed poorly compared with the SVM_1-4 models.

## Discussion

We investigated how glioma-induced microstructural alterations to WM were associated with functional motor deficits by mapping dMRI metrics along the CST. In segment-wise group comparison, we found significant differences between the ipsilesional tract profiles in the two patient groups (Class 0; Class 1) in relation to all dMRI metrics except for FD. The ipsilateral differences were mainly seen at the level of the glioma (superior portion of the tract), which showed the direct influence of the tumour area on motor function. However, tumour impact on tract metrics could also be detected in areas relatively distant from the tumour and peritumoural oedema especially in ADC and RD, demonstrating the spreading of the local tumour effect with respect to the motor status.

FA and ADC have been largely used to evaluate the WM, and both lower FA values and higher ADC values are associated with WM impairment. FA has been shown to be sensitive to detecting changes in WM water diffusion in cases of neuropathology and tends to decrease in areas where tumour cells have invaded WM.^86–88^ ADC indicates the M diffusivity of water molecules and an increase in ADC has been observed in pathologies accompanied by, e.g. oedema or necrosis^29,89^ and higher values are expected in voxels with low anisotropy. These metrics have been used to describe specific types of WM impairments, e.g. demyelination, axonal injury, inflammation or necrosis.^90,91^ However, these metrics may be affected by several factors^90,91^ and they might be less sensitive to distinguish specific types of WM impairments.^90,91^ The majority of our cohort with glioblastomas (GBMs; 77 patients) are characterized by diffuse infiltration into the normal brain. We observed the greatest elevation in values along the CST in relation to ADC, which could be explained by GBMs’ frequently developing within the WM and spreading throughout the brain along fibres,^92^ and limited diffusion because of their tissue properties. However, this explanation should be taken with caution. These results confirmed that the ipsilesional CST fibres were affected and correlated with the patient’s motor deficits.

Furthermore, AD and RD are measures of diffusion in perpendicular and parallel directions to the principal axis of diffusion, respectively. The incorporation of these metrics has been shown to lead to better differentiation between axonal injury or degeneration (AD) and pathological demyelination (RD).^29,89,93–95^ Myelin fragmentation results in an increase in RD since the myelin sheaths block water diffusion out of the axon.^96^ Demyelination could be more of a chronic/slow process, while axonal degeneration could be a more acute and potentially clinically more detrimental process.

Several studies in brain tumour patients investigated DTI metrics as a diagnostic and prognostic biomarker for motor function.^87,97–101^ Decreases in FA values were associated with preoperative motor deficits.^87,97,98^ Other studies showed that lower average FA values within the affected CST as well as higher average ADC values are significantly associated with postoperative motor deficits.^18^

Here, we did not see a strong improvement in our SVM model performance on basis of AD. However, the KU of tract profile based on RD was highly effective (Table 4). Besides, we even found differences in segments/regions along contralesional tract profiles with respect to ADC and AD, supporting the assumption that contralesional CST may play a role in the postoperative motor outcome and recovery of motor function. These results may have implications for the compensatory strategy of the brain.

### SVM analysis

As explained earlier, SVM has been used in various clinical applications such as tumour segmentation and classification,^40–43^prediction of tumour consistency,^46^ prediction of prognosis and survival time of tumour patients using multi-modal imaging.^35,47^

### Microstructural measures

Considering dMRI-based features within the ipsi- and contralesional CST, we successfully developed several SVM-based models to classify patients with respect to their motor deficits (Class 0; Class 1). The best model performance achieved an AUC of 76.6% (SVM_2). The most effective features were FA_KU, ADC_M, RD_KU, FA_SK and ADC_STD. Accordingly, ADC and FA were identified as the most relevant metrics which better accounted for the detection of motor function impairment.^102^ These findings are also in line with our previous studies.^16,18^ In Fekonja *et al*.,^16^ we found FA and ADC metrics to be the most relevant metrics for detecting CST impairments, and in Rosenstock *et al*.,^18^ we found that peritumoural ADC and FA were strongly associated with postoperative motor deficits (motor outcome).

Here, FD was used to better account for the fibre orientation-specific microstructural properties in relation to infiltrating tumours. CSD can distinguish complex fibre populations in the brain. CSD estimates FODs within each voxel, based on the expected signal from a single collinearly oriented fibre population.^28^ Modern, CSD-based FD and FBA methods offer promising opportunities since they are related to the intra-axonal restricted compartment that is specific to a certain fibre orientation within a voxel.^16,31^ However, the stronger contribution of FA and ADC to our SVM models showed that these metrics are more robust for detecting motor impairment than FD in typical, clinical single-shell *b* = 1000 dMRI sequences, but might perform better when using multi-shell dMRI data. This finding is consistent with our previous results where ADC and FA presented higher sensitivity to detect CST impairment in tumour patients than FD.^16^

The univariate analysis confirmed our SVM model results to some extent, showing the predictive power of each tract profile’s measure (input feature) separately. All models which were trained on each AD, ADC, FA and AD metric separately, reached a relatively high specificity though they dealt with low sensitivity. Among them, SVM_FA provides a higher sensitivity but lower specificity, and SVM_ADC and SVM_RD provided higher specificity. Combining these metrics (SVM_2) led to both high sensitivity and specificity. FA_SK and RD_KU were not significantly different between the two patient groups, although they were selected as two of the most effective features. Features identified as significantly relevant and/or predictively relevant can agree or diverge, and numerous studies have been conducted demonstrating the differences between highly predictive and highly significant variable sets.^103,104^ Sometimes a strong predictivity fails to be significant, as it only provides supplementary information and could increase the predictive power of ML models just in combination with other features. The SVM_2 model reached the best performance among all trained models which showed that the Mdn profile corresponded to better prediction accuracy in comparison with the Mahalanobis-based weighted M tract profiles—a method described in Yeatman *et al*.^15^ and Richie-Halford *et al*., that implies the robustness of the Mdn method in the specific case since outlier segments with extreme values do not bias the Mdn. The superiority of the Mdn profile has been previously shown in,^106^ where microstructural models were used to understand the role of WM in relation to cognitive development. Our SVM_1-4 models, which were based on the extracted features, were more efficient and robust compared with SVM_5 since these models were less complex in their design. The high dimensional dMRI data (100 values per tract profile) and low available number of patient data resulted in lower performance in SVM_5. The variety in glioma location in relation to the CST as well as the low sample size restricted our analysis when SVM_5 tried to capture all patterns of microstructural variations. Therefore, we were not able to detect all segment-wise variations as efficiently as possible in SVM_5. In the SVM_1-4 models, we were able to summarize the segment-wise information of dMRI-based CST profiles as different statistical measures to detect informative glioma-induced microstructural alterations to WM to predict functional motor deficits.

### Demographic and clinical features

The SVM model with only demographics and clinical variables (SVM_clinical), such as age, gender, glioma location, glioma WHO grade and RMT ratio, showed a poor performance (56% accuracy and 62% AUC) while the models with microstructural measures as input, e.g. SVM_2 (74% accuracy and 77% AUC), reached to relatively high performance. We further assessed the performance of the SVM_1-5 models integrating patient demographics and clinical features and saw that none of them affected the SVM models’ performances. This could indicate the lower effectiveness of these features compared with tract profile-based characteristics (microstructural measures). In addition, the dMRI-based measures could be associated with different variables. Since the patient’s age was significantly different between the two patient groups, it was expected to improve our predictive models’ performances in combination with the microstructural measures. However, the integration of patients’ age did not improve the models’ performances. The performed CCA showed a strong and significant correlation between dMRI extracted features and age. Interestingly, we found a strong and significant correlation in the reverse direction for both motor groups (Class 0; Class 1). This confirms previous findings^107–109^ in which WM changes in relation to age and its variation as a function of age were investigated. In a recent study, age has been accurately predicted by FA and ADC metrics.^105^ These results justify as well that if taking into account the microstructural measures, age is of critical importance in distinguishing between the two motor groups.

According to CCA analysis and our SVM results, we could conclude that the information provided with age was apparently sufficiently covered by dMRI-based extracted features and thus no additional information was found considering patients’ age.

In conclusion, SVM_2 was the best model, which showed that the median-based tract profiles were more accurate than the M-weighted tract profile. This might be due to the median being more robust to outliers. The inclusion of demographic and clinical features did not affect model performance. This means that the dMRI-based measures are more powerful than these features in this context. The SVM_5 model performed poorly compared with SVM_2, indicating that the patterns of segmental information could not be perfectly detected with PCA.

### Translational aspect

The body of evidence that preservation of the WM connectivity is a key to preserving function is steadily growing. Therefore, not only the presurgical assessment of the spatial relation of the tumour and the tracts but also a detailed analysis of the impact the tumour already exerts on the WM is of great importance. As demonstrated in this study, ML shows a promising potential to address the microstructural effects of brain tumours on the WM, which is not accessible with traditional statistical methods, since it allows for discovering patterns in dMRI data and well-approximating complex relationships. Future studies need to further correlate ML finding with functional outcomes to establish new biomarkers for WM resilience to surgical manipulation with the promise to become a powerful prognostic tool in future neurosurgery.

### Limitations

Tractography suffers from a wide range of limitations that make its routine use problematic.^16,110^ Tractograms contain both false-positive^111^ and false-negative^112^ streamlines. In addition, tractography cannot distinguish between afferent and efferent connections, and streamlines may terminate improperly,^58^ especially in the case of tumours and oedema. Furthermore, our results are atlas and tractography algorithm dependent, since other tractography methods or atlas choices would possibly result in different tractograms.^16^ The dMRI data used for this study consists of a typical clinical single-shell acquisition and is thus limited for FD measurements due to incomplete attenuation of apparent extra-axonal signal.^16,113^ Furthermore, all patients received preoperative steroids to reduce oedema, which may cause a confounding effect. However, there is evidence that oedema has no strong influence on tractography results.^18^ In addition, the British Medical Research Council (BMRC) motor status does not necessarily detect subtle or apractic motor deficits which might correlate with early tumour effects on the WM. Indeed, the main limitation of our study is the relatively small sample size we could include to perform the ML analysis. Moreover, we binarized motor deficits due to a low number of samples per class. This led to inaccuracy and affected the performance of our ML models. To develop models performing multiclass classification which could consider differences in the degree of motor power (MRC = 1, 2, 3, 4, 5), larger samples would be needed per class.

A reliable way to evaluate the final ML model is to split the data into training, validation and test sets. The training set is used for learning and fitting the model’s parameters, a validation set is used to tune the model’s hyperparameters, while the test set is kept as an unseen data set to assess the performance of the final tuned ML model. This procedure offers an unbiased robust estimate of real model performance.^114^ However, this approach typically requires a large number of subjects, which is difficult to recruit in a clinical cohort, because they usually consist of small sample sizes for many ML methods. Moreover, to overcome the problem of the curse of dimensionality, where increasing the number of features requires larger training data to define a generalizable model, a reasonable sample size for training the ML models is necessary. CV^84,115^ is a common solution to estimate the model’s performance in case of small sample size or when validation with a separate data set is not feasible. However, model selection without nested CV uses the same test data to tune the model’s hyperparameters and evaluate the model,^84^ which is known to yield overly optimistic scores.^80^ To this end, here we performed nested CV and bootstrapping methods to enhance the generalizability of the ML models as well as to prevent an over-optimistic performance estimate. Nested CV uses a series of training, validation and test splits and fits the model iteratively using a pair of nested loops. In the inner loop, an optimal set of model’s hyperparameters is found using methods such as grid search on each training set, and each set of hyperparameters is evaluated using *k*-fold CV. In the outer loop, generalization error is estimated by averaging test set scores over several data set splits. Moreover, bootstrap aggregating (bagging)^116^ is applied to reduce the variance of a given estimator which uses CV^85^ itself to select fine-tuning parameters, trading off bias and variance of the bootstrap sample-specific candidate estimators. Overall, in the nested CV, the model is trained only using the training data (with five-fold inner CV). But the reported model accuracies are obtained using separate test data sets (with 10-fold CV). Therefore, the final test scores are computed on a completely independent set of samples than the training data as shown in Fig. 1.

## Conclusion

In this study, we analysed dMRI-based metrics to assess microstructural WM changes in correlation with the motor status of patients with gliomas in the motor system. We successfully developed SVM models to predict motor deficits in a heterogenous multivariate data set. ADC, FA and RD were highly predictive dMRI metrics. Additionally, we showed that dMRI metrics are better predictors than demographic and clinical variables, such as age, glioma grade and RMT ratio. Careful selection and testing of ML modelling are mandatory to prevent over- or underfitting and misinterpretation of data.

## Supplementary Material

fcac141_Supplementary_Data

## Abbreviations

AD =: axial diffusivity
ADC =: apparent diffusion coefficient
BMRC=: British Medical Research Council
CCA =: canonical correlation analysis
CSD =: constrained spherical deconvolution
CST =: corticospinal tract
CV =: cross validation
dMRI =: diffusion MRI
DTI =: diffusion tensor imaging
FA =: fractional anisotropy
FBA =: fixel-based analysis
FD =: fibre density
FDR =: false discovery rate
FOD =: fibre orientation distribution
GBM =: glioblastoma
KNN =: k-nearest neighbour
KU =: kurtosis
M =: mean
Mdn =: median
ML =: machine learning
MRC =: medical research council
PCA =: principal component analysis
RD =: radial diffusivity
RFE =: recursive feature elimination
RMT =: resting motor threshold
ROI=: Region of interest
SK =: skewness
STD =: standard deviation
SVM =: support vector machine
TMS =: transcranial magnetic stimulation
WM =: white matter
