# Supplementary material for: Machine learning-based prediction of motor status in glioma patients using diffusion MRI metrics along the corticospinal tract
Source: Brain Commun. 2022 May 27;4(3):fcac141. doi: 10.1093/braincomms/fcac141 (PMC9175193; doi:10.1093/braincomms/fcac141)
Supplement: fcac141_Supplementary_Data [file fcac141_supplementary_data.pdf]

Supplementary Table 1: CCA correlation coefficients

| CCX_1 | CCY_1 |
|---|---|
| 0.3686912166385593 | 1.0104415505824895 |
| 0.10362663900566732 | -0.15370543189728422 |
| 0.04403315110177353 | 0.45900350624996517 |
| 0.03699685340390663 | -0.5213307947856338 |
| -0.17735774359011147 | -0.5826016886003588 |
| 0.14144572832025423 | 0.8266288691383148 |
| -0.14389351484917856 | -0.8889561576739835 |
| 0.027427938022573206 | -0.7664143700445336 |
| 0.0484294883680349 | -0.398789007156184 |
| -0.048689189391995125 | 0.09137814336161552 |
| 0.04167285754217588 | 1.3780669134708392 |
| 0.2590717825660527 | 1.1942542320266645 |
| -0.1472697320691347 | -0.46005990097090893 |
| 0.07562702673713945 | 0.09137814336161552 |
| -0.039559172595529324 | 0.33646171862051527 |
| -0.06293601384047992 | 1.0717124443972146 |
| -0.2553732760525167 | -1.5016650958212328 |
| -0.14105888518587928 | -1.0727688391181582 |
| -0.0473670145207456 | -1.317852414377058 |
| -0.3988483653797505 | -1.7467486710801325 |
| -0.13117284178465785 | -0.7664143700445336 |
| -0.42049385794971866 | -1.0114979453034334 |
| 0.2361710754481009 | 1.1942542320266645 |
| 0.18752567882663085 | 1.439337807285564 |
| -0.006205673042270071 | 0.64281618769414 |
| 0.04854366866083257 | -0.21497632571200917 |
| -0.051634421737504155 | -0.398789007156184 |
| 0.006099809021180888 | 0.7653579753235898 |
| -0.012605023879954158 | 0.64281618769414 |
| 0.2444290368590513 | 1.500608701100289 |
| 0.3569216704829967 | 1.500608701100289 |
| -0.16985814362474877 | -0.2762472195267341 |
| 0.3716031027967968 | 1.3167960196561141 |
| -0.1691482163357569 | 0.2139199309910654 |
| -0.05928120851037519 | -1.440394202006508 |
| -0.19763593014147318 | -0.8889561576739835 |
| -0.25902187628122353 | -1.5016650958212328 |
| 0.13981394649063342 | -0.031163644267834356 |
| -0.23389167446150771 | -1.5629359896359578 |
| -0.25864717821041683 | -0.398789007156184 |

| | |
|---|---|
| -0.2377550400139979 | -1.1340397329328833 |
| -0.30359358883485676 | -1.5016650958212328 |
| -0.032678460551597066 | -1.8080195648948576 |
| 0.03372427650683013 | 0.15264903717634046 |
| -0.1035217616529111 | 0.8266288691383148 |
| -0.11259671233882773 | -0.9502270514887085 |
| -0.143517620363094 | -2.053103140153757 |
| -0.21655465058404733 | -1.5016650958212328 |
| 0.017365049436355508 | -0.031163644267834356 |
| 0.13399513399736385 | 0.7040870815088649 |
| 0.016772346545457117 | 0.64281618769414 |
| -0.08828321690555402 | -0.398789007156184 |
| 0.12267800447446522 | -0.33751811334145904 |
| -0.008023121242003661 | 0.45900350624996517 |
| 0.3081178290546914 | 0.03010724954689058 |
| -0.09312951597811121 | -0.15370543189728422 |
| 0.09283923334000155 | 0.33646171862051527 |
| -0.15162597058252636 | -0.5826016886003588 |
| -0.16581789182903844 | 1.1329833382119394 |
| -0.413272748943199 | -1.7467486710801325 |
| -0.2971687550920949 | -1.317852414377058 |
| -0.2928975011682272 | -0.398789007156184 |
| -0.23481293758078048 | -0.46005990097090893 |
| 0.14615638714669485 | -0.09243453808255929 |
| 0.047844373811946506 | 0.7040870815088649 |
| -0.1883070682285135 | 0.2751908248057903 |
| -0.07081406263180283 | -1.440394202006508 |
| -0.21716385487415982 | -1.317852414377058 |
| -0.36461738008333017 | -1.5629359896359578 |
| 0.18697497053090434 | 1.6844213825444638 |
| -0.09963706492817292 | -0.2762472195267341 |
| -0.13256894516837506 | 0.09137814336161552 |
| 0.06837227386648927 | 0.15264903717634046 |
| 0.46042944838314914 | 1.6844213825444638 |
| 0.076918596282874 | 0.7653579753235898 |
| 0.08497298065577868 | 0.33646171862051527 |
| 0.28343681857808045 | 1.0104415505824895 |
| 0.1475016935762437 | 0.7653579753235898 |
| 0.21206897900667582 | 0.9491706567677647 |
| -0.06116526778040114 | 0.7653579753235898 |
| 0.24563959901547888 | 1.439337807285564 |
| -0.026158822161424622 | 0.7040870815088649 |
| -0.002682222031399069 | -0.5826016886003588 |

| | |
|---|---|
| 0.16262747409970063 | 0.7040870815088649 |
| 0.27634393941922125 | 0.64281618769414 |
| 0.2947311692851994 | -0.21497632571200917 |
| 0.26758830157586366 | 0.8266288691383148 |
| -0.3692862370625389 | -1.8080195648948576 |
| 0.2964645250518494 | -0.46005990097090893 |
| 0.048033742165981774 | -0.8889561576739835 |
| 0.021088377936743785 | 0.7653579753235898 |
| 0.12823268308729202 | 1.3780669134708392 |
| 0.07055791424051508 | -0.5826016886003588 |
| 0.15975435631499993 | -0.33751811334145904 |
| -0.08736568282474619 | -0.2762472195267341 |
| -0.06829483834326558 | -1.6242068834506826 |
| 0.008068885726550568 | 0.5202744000646901 |
| 0.08908208666674716 | -0.7664143700445336 |
| 0.22251321030151677 | 1.1329833382119394 |
| -0.17171582364615545 | -1.317852414377058 |
| -0.45049276407935457 | -1.1953106267476081 |
| 0.15036583286041327 | 1.3167960196561141 |
| 0.01902318444759854 | -0.031163644267834356 |
| 0.07770181943201215 | 0.45900350624996517 |
| 0.08816528027367487 | -0.15370543189728422 |
| 0.11689277973220724 | 1.8682340639886388 |
| 0.10109922838249685 | 0.03010724954689058 |
| 0.040047488629599615 | 0.9491706567677647 |
| 0.1029032984760647 | 1.1329833382119394 |
| -0.25296601879859976 | 0.03010724954689058 |
| -0.02141469372431091 | 0.09137814336161552 |
| 0.06388993725933284 | 0.2751908248057903 |
| -0.05071430452563272 | -1.1953106267476081 |
| 0.09693971124921125 | 1.561879594915014 |
| 0.06023449221208867 | 2.0520467454328135 |
| 0.5762751146592526 | 1.3780669134708392 |

Supplementary Table 2: Corrected *p*-values (FDR-BH) along ipsilesional CST profiles

| AD | ADC | FA | FD | RD |
|---|---|---|---|---|
| 0.011136856046762069 | 0.011462777718257914 | 0.27965843978691385 | 0.336694391190237 | 0.023952035688187028 |
| 0.016744609325347045 | 0.011462777718257914 | 0.8614222560019962 | 0.8274002614710084 | 0.02009184498865534 |
| 0.03015882120803534 | 0.017049460927474815 | 0.7461304467136712 | 0.9141143985149782 | 0.023928278183991784 |
| 0.03463002505999671 | 0.017049460927474815 | 0.4992714530269339 | 0.9141143985149782 | 0.02000370924149419 |
| 0.03463002505999671 | 0.01609853273759804 | 0.33362888576119026 | 0.9888779233376925 | 0.01934585148806892 |
| 0.02929096906969759 | 0.015070328030297207 | 0.2411192776556674 | 0.9982066951663279 | 0.02000370924149419 |
| 0.02929096906969759 | 0.016414059129873117 | 0.1882602269445827 | 0.9982066951663279 | 0.02301654840936339 |
| 0.03171839305313943 | 0.01609853273759804 | 0.16357062674222658 | 0.9627634760330447 | 0.023377814261011088 |
| 0.025874570423586082 | 0.011767707372551789 | 0.14071099944515375 | 0.9141143985149782 | 0.02026124322500367 |
| 0.02121149154014225 | 0.011462777718257914 | 0.13618760908001423 | 0.9141143985149782 | 0.018164815368189098 |
| 0.021243206088001957 | 0.011462777718257914 | 0.137593259106739 | 0.9539734530140437 | 0.018164815368189098 |
| 0.02282886248425515 | 0.011767707372551789 | 0.14071099944515375 | 0.9627634760330447 | 0.01890593775097565 |
| 0.02282886248425515 | 0.011767707372551789 | 0.16357062674222658 | 0.9732034434130821 | 0.02009184498865534 |
| 0.026836322510219602 | 0.011767707372551789 | 0.14071099944515375 | 0.9542175652766098 | 0.01890593775097565 |
| 0.03171839305313943 | 0.011767707372551789 | 0.13618760908001423 | 0.9542175652766098 | 0.018164815368189098 |
| 0.03171839305313943 | 0.011767707372551789 | 0.14071099944515375 | 0.9141143985149782 | 0.01871782297626124 |
| 0.03463002505999671 | 0.011462777718257914 | 0.13618760908001423 | 0.7993414709537926 | 0.018164815368189098 |
| 0.055242742382381436 | 0.01176252940160753 | 0.1123439735986235 | 0.7735097655761669 | 0.018164815368189098 |
| 0.06098530177861444 | 0.01637697785263732 | 0.12522090303602273 | 0.732829340561692 | 0.02049227013218372 |
| 0.056990371311264 | 0.02023806896489688 | 0.14379811183280242 | 0.670781882771652 | 0.023377814261011088 |
| 0.055242742382381436 | 0.019547520296756882 | 0.17010815741260482 | 0.6256598717427512 | 0.024336388411057005 |
| 0.06098530177861444 | 0.02323681379112752 | 0.21170746187000897 | 0.6042199620338617 | 0.029665195952593204 |
| 0.08994367978888763 | 0.0338046226196035 | 0.2943903786876607 | 0.6042199620338617 | 0.04359606908955554 |
| 0.12999433043589012 | 0.04208287858689789 | 0.3982503178400343 | 0.6042199620338617 | 0.06286515888821723 |
| 0.14699471121546623 | 0.03634301769573582 | 0.5425887833684939 | 0.6042199620338617 | 0.07156173355217792 |
| 0.15608847981541277 | 0.028789716935267145 | 0.6291283969128854 | 0.5822955665604527 | 0.06969162288672615 |
| 0.16651563180443216 | 0.026615777124084465 | 0.6012948757163232 | 0.4325106201914536 | 0.06691954796741748 |
| 0.17812251369044055 | 0.02917542685817119 | 0.5734108400585972 | 0.2819084518363021 | 0.06883391740536415 |
| 0.19923711247861567 | 0.02917542685817119 | 0.5326023926399954 | 0.19195509787067483 | 0.061758018317453554 |
| 0.2266382440708272 | 0.02832680226944813 | 0.4613677639298348 | 0.1485938230016407 | 0.04440187444481049 |
| 0.2266382440708272 | 0.023864080709273018 | 0.3982503178400343 | 0.1485938230016407 | 0.03468365486650289 |
| 0.21269741546085077 | 0.024831482722872774 | 0.38007842641007183 | 0.1485938230016407 | 0.03468365486650289 |
| 0.20946354884691123 | 0.027700280201350142 | 0.3896753234923075 | 0.1485938230016407 | 0.040900643477244016 |
| 0.17812251369044055 | 0.025011095415107754 | 0.38563100423617064 | 0.1485938230016407 | 0.04083623304439021 |
| 0.13581417513481156 | 0.018157248529293974 | 0.31670973161260846 | 0.1485938230016407 | 0.03291789003938622 |
| 0.11889043648836928 | 0.015956387876704864 | 0.2786671226853675 | 0.1485938230016407 | 0.025887432625942456 |
| 0.10458775476628153 | 0.012048039939190944 | 0.213333119674164 | 0.1485938230016407 | 0.023377814261011088 |
| 0.0795752340698011 | 0.011462777718257914 | 0.14492614860552588 | 0.1485938230016407 | 0.01934585148806892 |
| 0.05863356146822964 | 0.011462777718257914 | 0.1123439735986235 | 0.1485938230016407 | 0.018164815368189098 |
| 0.056990371311264 | 0.011462777718257914 | 0.07994206707289495 | 0.1485938230016407 | 0.018076665667520586 |

| | | | | |
|---|---|---|---|---|
| 0.05792775878183194 | 0.011462777718257914 | 0.05959326596819153 | 0.1485938230016407 | 0.017421757849957253 |
| 0.05401262186603804 | 0.011462777718257914 | 0.04550526922929115 | 0.1485938230016407 | 0.01588723194494714 |
| 0.04470889004892224 | 0.011462777718257914 | 0.04078425140010971 | 0.1485938230016407 | 0.01588723194494714 |
| 0.04470889004892224 | 0.011462777718257914 | 0.04078425140010971 | 0.1485938230016407 | 0.01588723194494714 |
| 0.04870401647324613 | 0.011462777718257914 | 0.04078425140010971 | 0.1485938230016407 | 0.017421757849957253 |
| 0.05863356146822964 | 0.011497714725035172 | 0.04078425140010971 | 0.1485938230016407 | 0.018164815368189098 |
| 0.07901994313636655 | 0.011767707372551789 | 0.04078425140010971 | 0.15226213864144128 | 0.018164815368189098 |
| 0.09306233751815475 | 0.012747849113972198 | 0.041863934899368835 | 0.17571266360674254 | 0.018164815368189098 |
| 0.10458775476628153 | 0.013619963168304565 | 0.04550526922929115 | 0.18890654634936266 | 0.018164815368189098 |
| 0.11777182152502443 | 0.014565874125948888 | 0.04740731515010825 | 0.18907617870645227 | 0.01890593775097565 |
| 0.11889043648836928 | 0.01609853273759804 | 0.05137638799979671 | 0.18907617870645227 | 0.02000370924149419 |
| 0.12078500583653652 | 0.017999557623633866 | 0.061543138659016054 | 0.19233816913746687 | 0.021779460615798985 |
| 0.11889043648836928 | 0.021012431943190846 | 0.07780416004578886 | 0.23259677308874643 | 0.023928278183991784 |
| 0.10458775476628153 | 0.023614098401770214 | 0.10144973516677312 | 0.27743331853289555 | 0.026462791433194653 |
| 0.07952113868114824 | 0.024431221390329605 | 0.12522090303602273 | 0.336694391190237 | 0.03157076588168193 |
| 0.05863356146822964 | 0.02323681379112752 | 0.14071099944515375 | 0.43735071883222243 | 0.03550853313730138 |
| 0.04773526620930219 | 0.02171390340272644 | 0.16654117355485182 | 0.478215731155554 | 0.0381613645019177 |
| 0.03463002505999671 | 0.017893599449023478 | 0.20009489545153625 | 0.6042199620338617 | 0.0381613645019177 |
| 0.024382381326636202 | 0.015070328030297207 | 0.22699672556737807 | 0.6859842391892909 | 0.03588214425467024 |
| 0.016744609325347045 | 0.011894922454589696 | 0.2411192776556674 | 0.7695288387565576 | 0.03313585756572504 |
| 0.01392081883561952 | 0.011497714725035172 | 0.2411192776556674 | 0.789757454540459 | 0.02851365755958812 |
| 0.011136856046762069 | 0.011462777718257914 | 0.2411192776556674 | 0.789757454540459 | 0.02566864216123481 |
| 0.011136856046762069 | 0.011462777718257914 | 0.2411192776556674 | 0.789757454540459 | 0.023928278183991784 |
| 0.011136856046762069 | 0.011462777718257914 | 0.2411192776556674 | 0.789757454540459 | 0.023278674419693245 |
| 0.011136856046762069 | 0.011462777718257914 | 0.2411192776556674 | 0.789757454540459 | 0.02301654840936339 |
| 0.011136856046762069 | 0.011462777718257914 | 0.25535977736224225 | 0.789757454540459 | 0.023278674419693245 |
| 0.011136856046762069 | 0.011462777718257914 | 0.2639165492491231 | 0.789757454540459 | 0.023928278183991784 |
| 0.011136856046762069 | 0.011462777718257914 | 0.2786671226853675 | 0.789757454540459 | 0.023952035688187028 |
| 0.011136856046762069 | 0.011462777718257914 | 0.27965843978691385 | 0.789757454540459 | 0.023952035688187028 |
| 0.011136856046762069 | 0.011462777718257914 | 0.2943903786876607 | 0.789757454540459 | 0.024732373214574433 |
| 0.011206775544242457 | 0.011462777718257914 | 0.31064768769730416 | 0.789757454540459 | 0.02518303307330361 |
| 0.01286524468623974 | 0.011462777718257914 | 0.31285805946128703 | 0.789757454540459 | 0.023952035688187028 |
| 0.014109536187488588 | 0.011462777718257914 | 0.30917252479607626 | 0.789757454540459 | 0.023928278183991784 |
| 0.014890466596799495 | 0.011462777718257914 | 0.27965843978691385 | 0.789757454540459 | 0.023278674419693245 |
| 0.018680309910050114 | 0.011462777718257914 | 0.25535977736224225 | 0.789757454540459 | 0.021394064999468276 |
| 0.02282886248425515 | 0.011462777718257914 | 0.22699672556737807 | 0.789757454540459 | 0.02000370924149419 |
| 0.02929096906969759 | 0.011462777718257914 | 0.16357062674222658 | 0.7695288387565576 | 0.019294243701493925 |
| 0.03463002505999671 | 0.011462777718257914 | 0.12522090303602273 | 0.6042199620338617 | 0.018164815368189098 |
| 0.04021560781878949 | 0.011497714725035172 | 0.07780416004578886 | 0.4658812642859192 | 0.018164815368189098 |
| 0.04470889004892224 | 0.011497714725035172 | 0.05088356958280326 | 0.3533492096113398 | 0.018164815368189098 |
| 0.04870401647324613 | 0.011497714725035172 | 0.04078425140010971 | 0.2770301593694052 | 0.018164815368189098 |
| 0.04870401647324613 | 0.011462777718257914 | 0.03245043014523583 | 0.23349455839247987 | 0.018033289115355795 |
| 0.04870401647324613 | 0.011462777718257914 | 0.02511995941577425 | 0.19195509787067483 | 0.01588723194494714 |

| | | | | |
|---|---|---|---|---|
| 0.04470889004892224 | 0.011462777718257914 | 0.020169973894517256 | 0.17571266360674254 | 0.015527430091551199 |
| 0.043107132670720964 | 0.011462777718257914 | 0.018137062558646628 | 0.15226213864144128 | 0.013917474296056631 |
| 0.0346300250599967 | 0.011462777718257914 | 0.01662788252002256 | 0.15074839122312966 | 0.013323800502485033 |
| 0.03155622017843677 | 0.011462777718257914 | 0.01662788252002256 | 0.1485938230016407 | 0.012202570484895169 |
| 0.025574079303818092 | 0.011462777718257914 | 0.01662788252002256 | 0.1485938230016407 | 0.012202570484895169 |
| 0.022622328713007275 | 0.011462777718257914 | 0.01662788252002256 | 0.1485938230016407 | 0.012202570484895169 |
| 0.019106282780761756 | 0.011462777718257914 | 0.01662788252002256 | 0.1485938230016407 | 0.012202570484895169 |
| 0.016744609325347045 | 0.011462777718257914 | 0.01662788252002256 | 0.15226213864144128 | 0.012202570484895169 |
| 0.014890466596799495 | 0.011462777718257914 | 0.01662788252002256 | 0.18890654634936266 | 0.012202570484895169 |
| 0.014890466596799495 | 0.011462777718257914 | 0.018137062558646628 | 0.23349455839247987 | 0.012202570484895169 |
| 0.01393676144721457 | 0.011462777718257914 | 0.02424750538883141 | 0.3317422608885178 | 0.012202570484895169 |
| 0.011882195455281816 | 0.011462777718257914 | 0.035301485609796034 | 0.47676095184626227 | 0.012202570484895169 |
| 0.011136856046762069 | 0.011462777718257914 | 0.04078425140010971 | 0.6473393205321074 | 0.012202570484895169 |
| 0.011136856046762069 | 0.011462777718257914 | 0.06075201010117749 | 0.789757454540459 | 0.012202570484895169 |
| 0.011136856046762069 | 0.011462777718257914 | 0.10144973516677312 | 0.8871531125264345 | 0.012202570484895169 |
| 0.011136856046762069 | 0.011462777718257914 | 0.14492614860552588 | 0.9627634760330447 | 0.012202570484895169 |
| 0.011136856046762069 | 0.011462777718257914 | 0.6906425930218448 | 0.789757454540459 | 0.018164815368189098 |

Supplementary Table 3: Corrected *p*-values (FDR-BH) along the differences between ipsi- and contralesional CST profiles

| AD | ADC | FA | FD | RD |
|---|---|---|---|---|
| 0.946710846397834 | 0.7807702814114579 | 0.37323011750235585 | 0.9775738965452768 | 0.5838802437081158 |
| 0.4818589402602446 | 0.556726936781187 | 0.8410825003874249 | 0.9040061752791977 | 0.5928992668593196 |
| 0.42558388377505574 | 0.29474932132860976 | 0.7166517983490064 | 0.8313145176230737 | 0.2686273630726987 |
| 0.3645473342101959 | 0.13266042076113374 | 0.3153792871266046 | 0.837624646639151 | 0.10380438744659091 |
| 0.3645473342101959 | 0.08866269318085154 | 0.18350084927364785 | 0.6495610369980465 | 0.07366599053088398 |
| 0.3735489881783121 | 0.1040775601787513 | 0.14168057428042644 | 0.6427869803426212 | 0.07634300862113903 |
| 0.4538768865847612 | 0.14407507449662693 | 0.11596421370663372 | 0.6328905221979035 | 0.10682710846045199 |
| 0.6588512122768341 | 0.1758508019417226 | 0.08669312988369528 | 0.6328905221979035 | 0.1278380980062686 |
| 0.751280416125299 | 0.21034427748509554 | 0.06482408763999176 | 0.6044532261016083 | 0.1278380980062686 |
| 0.7813800699684113 | 0.2979382908536028 | 0.06721042042700122 | 0.6328905221979035 | 0.15082288891412649 |
| 0.9034658406277295 | 0.6142184862401171 | 0.08669312988369528 | 0.6524646842379762 | 0.2686273630726987 |
| 0.8780239686427145 | 0.8968498784656392 | 0.12974703468049267 | 0.7553173382781562 | 0.4476555411542437 |
| 0.8469400277391879 | 0.9910317149864964 | 0.1553741744955487 | 0.8172452181173122 | 0.5291047581762206 |
| 0.7813800699684113 | 0.9602345364417547 | 0.15083470128058205 | 0.8155503794384331 | 0.5110852133591515 |
| 0.7813800699684113 | 0.9399810614796142 | 0.1553741744955487 | 0.9734637368410731 | 0.36904925261372795 |
| 0.8780239686427145 | 0.8788745710258984 | 0.19557810828249525 | 0.9775738965452768 | 0.3635930458206528 |
| 0.8780239686427145 | 0.756776441358386 | 0.2019855719955663 | 0.9734637368410731 | 0.3257185261513045 |
| 0.8780239686427145 | 0.6558705945376152 | 0.17995604433123588 | 0.9734637368410731 | 0.25840855408827007 |
| 0.9815595076030244 | 0.5975384727625117 | 0.18355486471250076 | 0.9734637368410731 | 0.26545028553115174 |
| 0.8780239686427145 | 0.5152732922843374 | 0.19322221671857648 | 0.9638786823435999 | 0.2402165384885349 |
| 0.8469400277391879 | 0.3735900802009081 | 0.18350084927364785 | 0.946826799458898 | 0.2010606427452123 |
| 0.8382341274883265 | 0.3368503135066111 | 0.17116087929412843 | 0.8809348111747528 | 0.1839953263949054 |
| 0.8469400277391879 | 0.4290329175270216 | 0.23067527627375958 | 0.9040061752791977 | 0.21890095410974983 |
| 0.875295214773319 | 0.4590821724197558 | 0.3120567807889906 | 0.9638786823435999 | 0.2402165384885349 |
| 0.7813800699684113 | 0.3533166992981862 | 0.3612151694221496 | 0.9899872919283637 | 0.21854516509838162 |
| 0.7388258758422597 | 0.21034427748509554 | 0.3203015998138497 | 0.9775738965452768 | 0.16149468799944056 |
| 0.6570449621119576 | 0.14407507449662693 | 0.22760500025472905 | 0.9495976721906235 | 0.1279680535406273 |
| 0.5275718685377806 | 0.1366278639994293 | 0.19761984533463753 | 0.7630581559197008 | 0.13944104288251719 |
| 0.4705315369348145 | 0.1131651695996264 | 0.18355486471250076 | 0.6704805735342625 | 0.1278380980062686 |
| 0.5030343148422163 | 0.09569297073289015 | 0.1553741744955487 | 0.6458222576050873 | 0.09361981768721692 |
| 0.4705315369348145 | 0.0831925205126628 | 0.1553741744955487 | 0.6458222576050873 | 0.08575717647527047 |
| 0.4538768865847612 | 0.09001828786424906 | 0.18350084927364785 | 0.643190672951719 | 0.09594313955215965 |
| 0.4538768865847612 | 0.1131651695996264 | 0.22945409490469965 | 0.6524646842379762 | 0.1278380980062686 |
| 0.4538768865847612 | 0.1131651695996264 | 0.2539560632242701 | 0.6704805735342625 | 0.1278380980062686 |
| 0.42558388377505574 | 0.08949225398151685 | 0.2019855719955663 | 0.6704805735342625 | 0.10982183469040709 |
| 0.4466102658776658 | 0.08295252674525776 | 0.18355486471250076 | 0.6795303498327848 | 0.09594533336998949 |
| 0.43468635751057527 | 0.08007254592202284 | 0.1553741744955487 | 0.6704805735342625 | 0.09309908885774293 |
| 0.42558388377505574 | 0.07085636337170394 | 0.13803205013157507 | 0.6524646842379762 | 0.07533978150946301 |

| | | | | |
|---|---|---|---|---|
| 0.4168535932962531 | 0.07085636337170394 | 0.09511219401924051 | 0.6458222576050873 | 0.053407816047747116 |
| 0.42558388377505574 | 0.07085636337170394 | 0.0721607634931837 | 0.6427869803426212 | 0.052370196524349044 |
| 0.4538768865847612 | 0.07085636337170394 | 0.06482408763999176 | 0.6427869803426212 | 0.052370196524349044 |
| 0.4705315369348145 | 0.07085636337170394 | 0.053400683202913836 | 0.6328905221979035 | 0.052370196524349044 |
| 0.4818589402602446 | 0.07085636337170394 | 0.053400683202913836 | 0.6328905221979035 | 0.052370196524349044 |
| 0.5071634137125404 | 0.07085636337170394 | 0.053400683202913836 | 0.6044532261016083 | 0.052370196524349044 |
| 0.514652407761254 | 0.07582894780604216 | 0.053400683202913836 | 0.6044532261016083 | 0.052370196524349044 |
| 0.6024072846192386 | 0.07901464834480984 | 0.053400683202913836 | 0.6044532261016083 | 0.052370196524349044 |
| 0.7194119106828601 | 0.08095735925122848 | 0.053400683202913836 | 0.6044532261016083 | 0.052370196524349044 |
| 0.7813800699684113 | 0.08125374404500925 | 0.053400683202913836 | 0.6044532261016083 | 0.052370196524349044 |
| 0.8469400277391879 | 0.08125374404500925 | 0.053400683202913836 | 0.6044532261016083 | 0.052370196524349044 |
| 0.8780239686427145 | 0.08125374404500925 | 0.053400683202913836 | 0.6044532261016083 | 0.052370196524349044 |
| 0.8780239686427145 | 0.0836934139524262 | 0.053400683202913836 | 0.6044532261016083 | 0.053407816047747116 |
| 0.8780239686427145 | 0.09116335398184212 | 0.053400683202913836 | 0.6044532261016083 | 0.05556674288447302 |
| 0.8780239686427145 | 0.10100102297715273 | 0.05533487826930164 | 0.6044532261016083 | 0.06250788708720481 |
| 0.875295214773319 | 0.1131651695996264 | 0.06482408763999176 | 0.6328905221979035 | 0.07239593478892002 |
| 0.7813800699684113 | 0.1131651695996264 | 0.08409221559866405 | 0.6328905221979035 | 0.08177527558658071 |
| 0.6570449621119576 | 0.10928301473566718 | 0.09717724750201813 | 0.6427869803426212 | 0.09086239954748633 |
| 0.514652407761254 | 0.09808096357351669 | 0.11796322308970694 | 0.6427869803426212 | 0.09175029282610467 |
| 0.464352857656369 | 0.08295252674525776 | 0.13715506819861734 | 0.6458222576050873 | 0.09105716260312426 |
| 0.42558388377505574 | 0.07644946202713564 | 0.14318687193700194 | 0.6495610369980465 | 0.08433823700610824 |
| 0.3645473342101959 | 0.07085636337170394 | 0.15083470128058205 | 0.6524646842379762 | 0.07555886579618676 |
| 0.35729119733545944 | 0.07085636337170394 | 0.15083470128058205 | 0.6704805735342625 | 0.07016266499101857 |
| 0.34112341487223097 | 0.07085636337170394 | 0.15083470128058205 | 0.6524646842379762 | 0.06456733653832186 |
| 0.34112341487223097 | 0.07085636337170394 | 0.15083470128058205 | 0.6495610369980465 | 0.0602467574325961 |
| 0.3538345288232321 | 0.07085636337170394 | 0.14318687193700194 | 0.6427869803426212 | 0.05518072776899828 |
| 0.3585798533491954 | 0.07085636337170394 | 0.12725758000572143 | 0.6427869803426212 | 0.053407816047747116 |
| 0.3585798533491954 | 0.07085636337170394 | 0.11596421370663372 | 0.6421060214145352 | 0.053407816047747116 |
| 0.36262055396206444 | 0.07085636337170394 | 0.10220940273130472 | 0.6328905221979035 | 0.053407816047747116 |
| 0.36262055396206444 | 0.07085636337170394 | 0.09717724750201813 | 0.6328905221979035 | 0.053407816047747116 |
| 0.3645473342101959 | 0.07085636337170394 | 0.09717724750201813 | 0.6328905221979035 | 0.053407816047747116 |
| 0.3645473342101959 | 0.07085636337170394 | 0.09360098287455311 | 0.6328905221979035 | 0.053407816047747116 |
| 0.3645473342101959 | 0.07085636337170394 | 0.0887404727518205 | 0.6427869803426212 | 0.053407816047747116 |
| 0.3735489881783121 | 0.07085636337170394 | 0.08366176387172798 | 0.6427869803426212 | 0.052370196524349044 |
| 0.4194614171345507 | 0.07085636337170394 | 0.07711797313590166 | 0.6524646842379762 | 0.052370196524349044 |
| 0.42558388377505574 | 0.07085636337170394 | 0.06721042042700122 | 0.6704805735342625 | 0.052370196524349044 |
| 0.4538768865847612 | 0.07085636337170394 | 0.06482408763999176 | 0.7553173382781562 | 0.052370196524349044 |
| 0.4538768865847612 | 0.07085636337170394 | 0.06482408763999176 | 0.7630581559197008 | 0.052370196524349044 |
| 0.464352857656369 | 0.07085636337170394 | 0.06482408763999176 | 0.7553173382781562 | 0.052370196524349044 |
| 0.4700769606431286 | 0.07582894780604216 | 0.05533487826930164 | 0.6704805735342625 | 0.052370196524349044 |
| 0.4705315369348145 | 0.07644946202713564 | 0.053400683202913836 | 0.6524646842379762 | 0.052370196524349044 |
| 0.464352857656369 | 0.07644946202713564 | 0.053400683202913836 | 0.6495610369980465 | 0.052370196524349044 |
| 0.464352857656369 | 0.07644946202713564 | 0.053400683202913836 | 0.6458222576050873 | 0.052370196524349044 |

| | | | | |
|---|---|---|---|---|
| 0.4538768865847612 | 0.0763728527061631 | 0.053400683202913836 | 0.6458222576050873 | 0.052370196524349044 |
| 0.4538768865847612 | 0.07085636337170394 | 0.053400683202913836 | 0.6427869803426212 | 0.052370196524349044 |
| 0.4297155255320146 | 0.07085636337170394 | 0.053400683202913836 | 0.6427869803426212 | 0.052370196524349044 |
| 0.42558388377505574 | 0.07085636337170394 | 0.053400683202913836 | 0.6328905221979035 | 0.052370196524349044 |
| 0.37909817365337545 | 0.07085636337170394 | 0.053400683202913836 | 0.6328905221979035 | 0.052370196524349044 |
| 0.3645473342101959 | 0.07085636337170394 | 0.053400683202913836 | 0.6328905221979035 | 0.052370196524349044 |
| 0.36262055396206444 | 0.07085636337170394 | 0.053400683202913836 | 0.6044532261016083 | 0.052370196524349044 |
| 0.3585798533491954 | 0.07085636337170394 | 0.04490255045469805 | 0.6044532261016083 | 0.047205873794830336 |
| 0.3538345288232321 | 0.06476818068785488 | 0.040204414262693715 | 0.6044532261016083 | 0.037394482643962355 |
| 0.34112341487223097 | 0.056976518189368115 | 0.040204414262693715 | 0.6044532261016083 | 0.03001955369125982 |
| 0.27764469545794945 | 0.05363070285608441 | 0.040204414262693715 | 0.6044532261016083 | 0.02885877403778891 |
| 0.27764469545794945 | 0.05237320079667769 | 0.040204414262693715 | 0.6044532261016083 | 0.02885877403778891 |
| 0.24466784630296584 | 0.04968901214595009 | 0.040204414262693715 | 0.6044532261016083 | 0.02885877403778891 |
| 0.2139348000837406 | 0.04968901214595009 | 0.040204414262693715 | 0.6044532261016083 | 0.02885877403778891 |
| 0.2139348000837406 | 0.04968901214595009 | 0.040204414262693715 | 0.6044532261016083 | 0.02885877403778891 |
| 0.2139348000837406 | 0.04968901214595009 | 0.04490255045469805 | 0.6389952771571914 | 0.02885877403778891 |
| 0.2139348000837406 | 0.04968901214595009 | 0.053400683202913836 | 0.6427869803426212 | 0.02885877403778891 |
| 0.2139348000837406 | 0.04968901214595009 | 0.053400683202913836 | 0.6458222576050873 | 0.02885877403778891 |
| 0.24466784630296584 | 0.07085636337170394 | 0.18355486471250076 | 0.7366544734706596 | 0.052370196524349044 |

Supplementary Table 4: Corrected *p*-values (FDR-BH) along contralesional CST profiles

| AD | ADC | FA | FD | RD |
|---|---|---|---|---|
| 0.02508361401551678 | 0.04154555678348011 | 0.997163526242788 | 0.8779638730808292 | 0.17545193564158262 |
| 0.059603828717824596 | 0.06248168193790106 | 0.997163526242788 | 0.9361604877288705 | 0.29124966338597363 |
| 0.12866833514902395 | 0.12481260112563006 | 0.997163526242788 | 0.9361604877288705 | 0.3640159192490626 |
| 0.17769372168447067 | 0.15934085421190475 | 0.997163526242788 | 0.9361604877288705 | 0.3721497560025711 |
| 0.17769372168447067 | 0.20645238225850812 | 0.997163526242788 | 0.9361604877288705 | 0.4014469495497067 |
| 0.13567001036343806 | 0.1752691719628336 | 0.997163526242788 | 0.9361604877288705 | 0.40557508684163673 |
| 0.08535688893399924 | 0.14174864986661165 | 0.997163526242788 | 0.9361604877288705 | 0.3813222472041167 |
| 0.04634553204449935 | 0.09339128552148872 | 0.997163526242788 | 0.9361604877288705 | 0.36686830084271066 |
| 0.04040866408314997 | 0.05990586893963859 | 0.997163526242788 | 0.9361604877288705 | 0.3640159192490626 |
| 0.04040866408314997 | 0.05108090109418563 | 0.997163526242788 | 0.9361604877288705 | 0.32349653047927374 |
| 0.027934404660784742 | 0.03707540919180264 | 0.997163526242788 | 0.9361604877288705 | 0.2449643400661747 |
| 0.013738437199972845 | 0.02486524452136699 | 0.997163526242788 | 0.9406509592269419 | 0.19875591240402657 |
| 0.009386995259569693 | 0.018307499820251205 | 0.997163526242788 | 0.9500814708197131 | 0.17545193564158262 |
| 0.006525497020498873 | 0.013427991933762175 | 0.997163526242788 | 0.9725931029062184 | 0.17545193564158262 |
| 0.006525497020498873 | 0.012835202438286843 | 0.997163526242788 | 0.9725931029062184 | 0.17545193564158262 |
| 0.006525497020498873 | 0.012835202438286843 | 0.997163526242788 | 0.9361604877288705 | 0.17545193564158262 |
| 0.006525497020498873 | 0.012835202438286843 | 0.997163526242788 | 0.9361604877288705 | 0.17545193564158262 |
| 0.009386995259569693 | 0.012835202438286843 | 0.997163526242788 | 0.9361604877288705 | 0.17545193564158262 |
| 0.02508361401551678 | 0.012835202438286843 | 0.997163526242788 | 0.9361604877288705 | 0.17545193564158262 |
| 0.040195240097377676 | 0.012835202438286843 | 0.997163526242788 | 0.9361604877288705 | 0.17545193564158262 |
| 0.04040866408314997 | 0.012835202438286843 | 0.997163526242788 | 0.9361604877288705 | 0.1996466731924586 |
| 0.04040866408314997 | 0.012835202438286843 | 0.997163526242788 | 0.9361604877288705 | 0.262713118884425 |
| 0.05128594475090856 | 0.012835202438286843 | 0.997163526242788 | 0.9361604877288705 | 0.31311600287936303 |
| 0.09316998561305778 | 0.01317613120760435 | 0.997163526242788 | 0.9361604877288705 | 0.35041665824668017 |
| 0.17769372168447067 | 0.018307499820251205 | 0.997163526242788 | 0.9361604877288705 | 0.4014469495497067 |
| 0.2487915492081619 | 0.038468424877019285 | 0.997163526242788 | 0.9302496766802602 | 0.5362131362882816 |
| 0.3657498244745791 | 0.05426667000979107 | 0.997163526242788 | 0.9302496766802602 | 0.6105570633562066 |
| 0.5184628960553053 | 0.08027769595160855 | 0.997163526242788 | 0.9302496766802602 | 0.6054715076988493 |
| 0.6628476104300701 | 0.13599162417326016 | 0.997163526242788 | 0.9302496766802602 | 0.6054715076988493 |
| 0.696209181836311 | 0.21695119591871875 | 0.997163526242788 | 0.8779638730808292 | 0.6554084093024616 |
| 0.7504931788809532 | 0.27504171925320003 | 0.997163526242788 | 0.6307515293453646 | 0.6554084093024616 |
| 0.7821489085475621 | 0.2587582140647035 | 0.997163526242788 | 0.5909523881329235 | 0.6105570633562066 |
| 0.7630396533256533 | 0.19524777399773294 | 0.997163526242788 | 0.41545920868590525 | 0.5580310705334856 |
| 0.7023449366135487 | 0.16409850340262336 | 0.997163526242788 | 0.41545920868590525 | 0.5362131362882816 |
| 0.6628476104300701 | 0.15761772343199912 | 0.997163526242788 | 0.41545920868590525 | 0.5164692446748043 |
| 0.5336839379121917 | 0.13544991392075362 | 0.997163526242788 | 0.41545920868590525 | 0.49422056983880175 |
| 0.4816473759146378 | 0.09879143754941584 | 0.997163526242788 | 0.41545920868590525 | 0.4155501010256586 |
| 0.3847311140607384 | 0.07663701747720182 | 0.997163526242788 | 0.5909523881329235 | 0.3813222472041167 |
| 0.3226998719660713 | 0.07226908783123424 | 0.997163526242788 | 0.6307515293453646 | 0.39316548694574266 |
| 0.24467576282988668 | 0.08027769595160855 | 0.997163526242788 | 0.6932351414388339 | 0.408139550038619 |

| | | | | |
|---|---|---|---|---|
| 0.16157707244488012 | 0.05990586893963859 | 0.997163526242788 | 0.6932351414388339 | 0.3721497560025711 |
| 0.09889316748987668 | 0.053660230569808856 | 0.997163526242788 | 0.7714928342891748 | 0.3263398595977217 |
| 0.06277109779590523 | 0.05108090109418563 | 0.997163526242788 | 0.9273232746747789 | 0.32349653047927374 |
| 0.05128594475090856 | 0.047852126291642566 | 0.997163526242788 | 0.9361604877288705 | 0.3263398595977217 |
| 0.0513365028037749 | 0.04593236990183122 | 0.997163526242788 | 0.9361604877288705 | 0.35041665824668017 |
| 0.050119872307892915 | 0.04593236990183122 | 0.997163526242788 | 0.9361604877288705 | 0.3721497560025711 |
| 0.04530941301465678 | 0.04798017468586765 | 0.997163526242788 | 0.9361604877288705 | 0.3813222472041167 |
| 0.04040866408314997 | 0.05042394193240203 | 0.997163526242788 | 0.9361604877288705 | 0.39650411846494826 |
| 0.04040866408314997 | 0.05108090109418563 | 0.997163526242788 | 0.9361604877288705 | 0.4137044559664638 |
| 0.04040866408314997 | 0.053660230569808856 | 0.997163526242788 | 0.9361604877288705 | 0.4887119110797641 |
| 0.04040866408314997 | 0.05374928487072884 | 0.997163526242788 | 0.9361604877288705 | 0.49985048500221185 |
| 0.04040866408314997 | 0.05393602559149837 | 0.997163526242788 | 0.9361604877288705 | 0.5164692446748043 |
| 0.04040866408314997 | 0.05381185041731105 | 0.997163526242788 | 0.9361604877288705 | 0.5362131362882816 |
| 0.04040866408314997 | 0.053660230569808856 | 0.997163526242788 | 0.9361604877288705 | 0.5362131362882816 |
| 0.04040866408314997 | 0.05374928487072884 | 0.997163526242788 | 0.9361604877288705 | 0.5362131362882816 |
| 0.04040866408314997 | 0.055676626492493775 | 0.997163526242788 | 0.9361604877288705 | 0.5362131362882816 |
| 0.04040866408314997 | 0.06006425256430102 | 0.997163526242788 | 0.9406509592269419 | 0.5580310705334856 |
| 0.04040866408314997 | 0.06248168193790106 | 0.997163526242788 | 0.9725931029062184 | 0.5580310705334856 |
| 0.04040866408314997 | 0.06248168193790106 | 0.997163526242788 | 0.9967038674411898 | 0.6105570633562066 |
| 0.04040866408314997 | 0.06248168193790106 | 0.997163526242788 | 0.9725931029062184 | 0.6542199574396032 |
| 0.04040866408314997 | 0.05972922431752787 | 0.997163526242788 | 0.9672952422724697 | 0.6542199574396032 |
| 0.04040866408314997 | 0.053660230569808856 | 0.997163526242788 | 0.9361604877288705 | 0.6542199574396032 |
| 0.04040866408314997 | 0.04593236990183122 | 0.997163526242788 | 0.9361604877288705 | 0.6542199574396032 |
| 0.04040866408314997 | 0.038468424877019285 | 0.997163526242788 | 0.9361604877288705 | 0.6542199574396032 |
| 0.04040866408314997 | 0.03707540919180264 | 0.997163526242788 | 0.9361604877288705 | 0.6612825989736123 |
| 0.04040866408314997 | 0.039864020271848946 | 0.997163526242788 | 0.9361604877288705 | 0.738189992483967 |
| 0.04040866408314997 | 0.04254875093717367 | 0.997163526242788 | 0.9361604877288705 | 0.7430024543639498 |
| 0.04040866408314997 | 0.04495889071293446 | 0.997163526242788 | 0.9361604877288705 | 0.7430024543639498 |
| 0.04040866408314997 | 0.04495889071293446 | 0.997163526242788 | 0.9361604877288705 | 0.7430024543639498 |
| 0.04040866408314997 | 0.0474209604883618 | 0.997163526242788 | 0.9361604877288705 | 0.7755789395855308 |
| 0.04040866408314997 | 0.05050370308502325 | 0.997163526242788 | 0.9361604877288705 | 0.8256487414292686 |
| 0.04040866408314997 | 0.04813357299126024 | 0.997163526242788 | 0.9361604877288705 | 0.8256487414292686 |
| 0.04040866408314997 | 0.04479282937833091 | 0.997163526242788 | 0.9361604877288705 | 0.8256487414292686 |
| 0.04040866408314997 | 0.03897089698142747 | 0.997163526242788 | 0.9406509592269419 | 0.7616657771871289 |
| 0.04040866408314997 | 0.03592798730561081 | 0.997163526242788 | 0.9725931029062184 | 0.7176163379375933 |
| 0.04040866408314997 | 0.03433693967860302 | 0.997163526242788 | 0.9967038674411898 | 0.6542199574396032 |
| 0.04505605887190529 | 0.03143515284630125 | 0.997163526242788 | 0.9725931029062184 | 0.5580310705334856 |
| 0.050119872307892915 | 0.027610229835800366 | 0.997163526242788 | 0.9406509592269419 | 0.46025006986953804 |
| 0.055790870869468524 | 0.02486524452136699 | 0.997163526242788 | 0.9361604877288705 | 0.3721497560025711 |
| 0.06836518646923101 | 0.021168584753493824 | 0.997163526242788 | 0.9361604877288705 | 0.32205353664946557 |
| 0.08519104622689083 | 0.018307499820251205 | 0.997163526242788 | 0.9361604877288705 | 0.2449643400661747 |
| 0.08519104622689083 | 0.015944395674716692 | 0.997163526242788 | 0.9361604877288705 | 0.1996466731924586 |
| 0.08264746361101617 | 0.013427991933762175 | 0.997163526242788 | 0.9361604877288705 | 0.17545193564158262 |

| | | | | |
|---|---|---|---|---|
| 0.08519104622689083 | 0.01317613120760435 | 0.997163526242788 | 0.9361604877288705 | 0.17545193564158262 |
| 0.08519104622689083 | 0.012835202438286843 | 0.997163526242788 | 0.9361604877288705 | 0.17545193564158262 |
| 0.08073723324089202 | 0.012835202438286843 | 0.997163526242788 | 0.9361604877288705 | 0.17545193564158262 |
| 0.05625367927398639 | 0.012835202438286843 | 0.997163526242788 | 0.9361604877288705 | 0.17545193564158262 |
| 0.046229397014786865 | 0.012835202438286843 | 0.997163526242788 | 0.9361604877288705 | 0.17545193564158262 |
| 0.04040866408314997 | 0.013427991933762175 | 0.997163526242788 | 0.9361604877288705 | 0.17545193564158262 |
| 0.04040866408314997 | 0.019954282921918313 | 0.997163526242788 | 0.9361604877288705 | 0.17545193564158262 |
| 0.04040866408314997 | 0.027610229835800366 | 0.997163526242788 | 0.9361604877288705 | 0.1996466731924586 |
| 0.04040866408314997 | 0.037210787258562444 | 0.997163526242788 | 0.9406509592269419 | 0.2449643400661747 |
| 0.046064684490059175 | 0.04466276051681297 | 0.997163526242788 | 0.9725931029062184 | 0.2622803489214446 |
| 0.05128594475090856 | 0.05042394193240203 | 0.997163526242788 | 0.9725931029062184 | 0.3183832438036129 |
| 0.05128594475090856 | 0.0553020677775697 | 0.997163526242788 | 0.9725931029062184 | 0.3721497560025711 |
| 0.04963854821753421 | 0.05764758038756385 | 0.997163526242788 | 0.9406509592269419 | 0.408139550038619 |
| 0.046229397014786865 | 0.05972922431752787 | 0.997163526242788 | 0.9361604877288705 | 0.46025006986953804 |
| 0.059603828717824596 | 0.15401863221958004 | 0.997163526242788 | 0.9361604877288705 | 0.6054715076988493 |
| 0.1493701218710107 | 0.5402929602430806 | 0.997163526242788 | 0.9361604877288705 | 0.8354764540704432 |
| 0.04040866408314997 | 0.2558138971405256 | 0.997163526242788 | 0.9361604877288705 | 0.6542199574396032 |

Supplementary Table 5a:

*p*-values for histogram-based features of ipsilesional tract profiles (M, STD, KU and SK)

| | Mean | Standard_deviation | Kurtosis | Skewness |
|---|---|---|---|---|
| **AD** | | | | |
| **Pvalue** | 0.00035915874512317827 | 0.2341572786697913 | 0.366947543185421 | 0.36481618144418837 |
| **Statistic** | 1000.0 | 1469.0 | 1537.0 | 1536.0 |
| **ADC** | | | | |
| **Pvalue** | 0.00020793562410994644 | 0.0006843192729760747 | 0.06445201298720575 | 0.38847701594380935 |
| **Statistic** | 974.0 | 1032.0 | 1329.0 | 1547.0 |
| **FA** | | | | |
| **Pvalue** | 0.015003176742231243 | 0.347920379642365 | 0.0007117261438705842 | 0.1485877267971279 |
| **Statistic** | 1214.0 | 1528.0 | 1034.0 | 1413.0 |
| **FD** | | | | |
| **Pvalue** | 0.07190077569372236 | 0.2482846512862143 | 0.36481618144418837 | 0.08694737148719373 |
| **Statistic** | 1339.0 | 1477.0 | 1536.0 | 1357.0 |
| **RD** | | | | |
| **Pvalue** | 0.00038203487598156724 | 0.001288235588776543 | 0.10629430993736705 | 0.4125591732724999 |
| **Statistic** | 1003.0 | 1065.0 | 1377.0 | 1558.0 |

Supplementary Table 5b:

p-values for histogram-based features of contralesional tract profiles (M, STD, KU and SK)

| | Mean | Standard_deviation | Kurtosis | Skewness |
|---|---|---|---|---|
| **AD** | | | | |
| **Pvalue** | 0.0029473206024855916 | 0.10944566682997958 | 0.3190837409993945 | 0.11375107562409548 |
| **Statistic** | 1111.0 | 1380.0 | 1514.0 | 1384.0 |
| **ADC** | | | | |
| **Pvalue** | 0.001288235588776543 | 0.0816905028870411 | 0.14085083793248715 | 0.09151784128287349 |
| **Statistic** | 1065.0 | 1351.0 | 1407.0 | 1362.0 |
| **FA** | | | | |
| **Pvalue** | 0.4302883040679208 | 0.3030707431663115 | 0.10525856321503085 | 0.3375093416727035 |
| **Statistic** | 1566.0 | 1506.0 | 1376.0 | 1523.0 |
| **FD** | | | | |
| **Pvalue** | 0.47966582022590476 | 0.30505263433040164 | 0.18534049210053155 | 0.02632941924235198 |
| **Statistic** | 1588.0 | 1507.0 | 1439.0 | 1255.0 |
| **RD** | | | | |
| **Pvalue** | 0.016339233313890682 | 0.05018258478215847 | 0.14727888645121695 | 0.12857297507759896 |
| **Statistic** | 1220.0 | 1307.0 | 1412.0 | 1397.0 |
